# Baseline Tumor Transcriptional Features and Event-Free Survival After Anti-CD19 CAR T-Cell Therapy with Axicabtagene Ciloleucel in Large B-Cell Lymphoma: An Exploratory Reanalysis of the Randomized ZUMA-7 Trial

**DOI:** 10.3390/medsci14060671

**Published:** 2026-10-13

**Authors:** Jean Silva de Souza Resende, Igor Samesima Giner, Maria Gabriela Custodio de Figueiredo, Mariana Martins Paraízo, Enzo Lalli, Mauro Antônio Alves Castro, Bonald Cavalcante de Figueiredo

**Affiliations:** 1 Instituto de Pesquisa Pelé Pequeno Príncipe, Curitiba 80250-060, PR, Braziligor.giner@professor.fpp.edu.br (I.S.G.); gabi.figueiredo@mail.utoronto.ca (M.G.C.d.F.); mariana.paraizo@pelepequenoprincipe.org.br (M.M.P.); 2 Faculdades Pequeno Príncipe, Curitiba 80230-020, PR, Brazil; 3 Bioinformatics and Systems Biology Laboratory, Federal University of Paraná, Curitiba 81531-980, PR, Brazil; 4 Institut de Pharmacologie Moléculaire et Cellulaire CNRS UMR7275, Inserm U1323, Université Côte d’Azur, 06560 Valbonne, France; lalli@ipmc.cnrs.fr

**Keywords:** large B-cell lymphoma, ZUMA-7, CAR T-cell therapy, axi-cel, transcriptomics, tumor microenvironment, treatment resistance, biomarkers, gene-by-treatment interaction

## Abstract

**Background/Objectives**: Anti-CD19 chimeric antigen receptor (CAR) T-cell therapy with axicabtagene ciloleucel (axi-cel) improves outcomes in relapsed/refractory large B-cell lymphoma (LBCL), but many patients relapse despite retained CD19 expression. We explored baseline tumor transcriptional features associated with event-free survival (EFS) after axi-cel. **Methods**: We reanalyzed baseline NanoString IO 360 profiles from the randomized ZUMA-7 trial (axi-cel, *n* = 134; standard of care [SOC], *n* = 122) using differential expression, gene set enrichment, MCP-counter deconvolution, Kaplan–Meier and Cox models adjusted for tumor burden and cell of origin, and gene-by-treatment interaction models. **Results**: T-cell exhaustion and HLA transcripts were not associated with response in either arm, and no transcript passed FDR correction in differential expression. In the axi-cel arm, 13 of 14 modeled transcripts remained associated with EFS after adjustment; eight (*MS4A1*, *CD19*, *TNFRSF17*, *HLA-DOB*, *CD45RA*, *DUSP5*, *KLRD1* and *WNT11*) were FDR-supported at every survival-analysis stage within the 25 prioritized candidates, but not panel-wide. Consistent with the primary ZUMA-7 report, higher B-cell transcript expression was associated with longer EFS. Interaction testing gave borderline support for a differential association between arms only for *KLRD1* and *DUSP5* (FDR = 0.049), robust to arm-stratified models; other candidates showed no or only nominal specificity. Apart from the B-lineage score (FDR = 0.029), no estimated immune or stromal population was associated with response. **Conclusions**: Baseline B-cell transcriptional programs and a small set of gene-level candidates are associated with EFS after axi-cel in ZUMA-7. These exploratory findings do not establish predictive value and require independent validation.

## 1. Introduction

Diffuse large B-cell lymphoma (DLBCL), the most frequent subtype of large B-cell lymphoma (LBCL), is the most common aggressive non-Hodgkin lymphoma in adults and is characterized by substantial biological and clinical heterogeneity. Despite major advances with first-line rituximab-based chemoimmunotherapy (R-CHOP), approximately 30–40% of patients develop relapsed or refractory (r/r) disease, which has historically been associated with a dismal prognosis and limited treatment options [1,2]. Following failure of first-line therapy, CD19-directed chimeric antigen receptor (CAR) T-cell therapies, including axicabtagene ciloleucel (axi-cel), have transformed the therapeutic landscape by inducing durable remissions in a subset of patients [3,4].

However, a substantial proportion of patients treated with axi-cel either fail to achieve a sustained complete response or experience early relapse. Although antigen escape through loss or reduced expression of CD19 has been described, disease progression frequently occurs despite preserved target expression, indicating that resistance is often mediated by mechanisms beyond antigen loss [5,6]. These observations support a model in which axi-cel resistance arises from a complex interplay between tumor-intrinsic biological programs and the surrounding tumor microenvironment (TME).

In conventional tumor immunology, immune evasion is frequently attributed to pre-existing T-cell exhaustion and impaired antigen-presentation machinery, including downregulation of human leukocyte antigen (HLA) molecules. Although pathways such as PD-1/PD-L1 signaling may contribute to adaptive resistance after CAR T-cell infusion, baseline global exhaustion signatures have not consistently stratified treatment responses independently [7,8]. Similarly, while immunosuppressive cellular populations have been associated with impaired CAR T-cell expansion and activity, quantitative estimates of immune and stromal infiltration alone do not fully account for the marked variability in clinical outcomes [9,10]. This discrepancy suggests that the functional state and phenotypic plasticity of malignant B cells may constitute more consequential barriers to CAR T-cell activity than the abundance of inhibitory cell populations alone.

Effective antitumor immunity also depends on the coordinated activation of inflammatory pathways. Acute activation of interferon (IFN) enhances target vulnerability, whereas an inability to mount an effective baseline inflammatory program, or its chronic dysregulation, can limit CAR T-cell engagement [11,12]. In large B-cell lymphoma specifically, tumor interferon signaling and suppressive myeloid cells have been associated with CAR T-cell failure [10]. Beyond classical immune signaling, emerging paradigms highlight the role of tumor dedifferentiation and aggressive tissue remodeling. Programs analogous to epithelial–mesenchymal transition-like (EMT-like) and alterations in cellular adhesion confer physical and biological protection against effector immune cell infiltration and cytotoxicity, representing a largely unexplored frontier in cellular immunotherapy resistance [13,14,15].

Conversely, preservation of mature B-cell lineage identity may favor therapeutic sensitivity. Sustained expression of canonical B-cell markers, including *CD19* and *MS4A1*, together with lineage-defining transcriptional regulators such as IRF8, may maintain a differentiated cellular state that is more permissive to immune recognition and elimination [16,17,18,19]. Thus, the balance between B-cell lineage integrity and the acquisition of inflammatory or tissue-remodeling features may influence the response to CD19-directed CAR T-cell therapy.

The primary translational analysis of ZUMA-7 has already shown that B-cell-related gene expression features and CD19 expression in pretreatment tumors were associated with event-free survival after axi-cel, but not after second-line standard-of-care therapy, and that the tumor microenvironment influenced the efficacy of both strategies [20]; comparable immune-contexture determinants were described in ZUMA-1 [21]. The B-cell lineage association is therefore not a new finding, and in the present work it is treated as an internal positive control. The open questions are whether individual genes that were not highlighted in the primary reports carry additional information, and whether their associations genuinely differ between the randomized arms. This distinction matters clinically, because the growing number of CD19-directed agents-CAR T cells, monoclonal antibodies, and antibody–drug conjugates-makes the sequencing of these therapies, and the resistance mechanisms that should inform it, an active area of investigation [22].

Here, we performed an exploratory secondary analysis of baseline targeted transcriptomic data from the randomized ZUMA-7 trial. We evaluated B-cell lineage integrity, canonical T-cell exhaustion markers, and the HLA antigen-presentation machinery; screened candidate transcripts for association with event-free survival; and used the randomized standard-of-care arm to test formally, through gene-by-treatment interaction models, whether candidate associations differed by treatment. The TCGA-DLBC cohort was used only as a descriptive external reference, and data from the single-arm ZUMA-1 trial were not included, for the reasons detailed in Section 2.1. All findings are presented as hypothesis-generating.

## 2. Materials and Methods

### 2.1. Study Design and Data Sources

This retrospective, exploratory secondary analysis used publicly available transcriptomic and clinical data from the randomized phase 3 ZUMA-7 trial (NCT03391466, https://clinicaltrials.gov/study/NCT03391466; accessed on 8 October 2026), in which patients with large B-cell lymphoma that was refractory to, or had relapsed within 12 months after, first-line chemoimmunotherapy were randomized to axi-cel or to standard-of-care (SOC) second-line therapy [3]. Baseline (pretreatment) tumor profiles were available for 256 patients: 134 in the axi-cel arm and 122 in the SOC arm. ZUMA-7 enrolled patients with large B-cell lymphoma (LBCL), including DLBCL not otherwise specified, high-grade B-cell lymphoma, and transformed follicular lymphoma; the term LBCL is therefore used for ZUMA-7 patients, whereas DLBCL is retained for TCGA-DLBC and for statements derived from the DLBCL literature.

For response-based comparisons, patients were classified as having a sustained response or treatment failure, corresponding to the “Ongoing” and “Others” categories defined in the primary ZUMA-7 translational analysis [20]. In the axi-cel arm, 58 patients had a sustained response, 70 had treatment failure, and 6 were unclassified; in the SOC arm, the corresponding numbers were 18, 80, and 24 (response unavailable). Unclassified patients and those with unavailable response were excluded from response-based comparisons but retained in the event-free survival (EFS) analysis. Baseline tumor burden (sum of product diameters, SPD) and cell of origin (COO) were extracted as clinical covariates.

The TCGA-DLBC cohort (https://portal.gdc.cancer.gov/projects/TCGA-DLBC; *N* = 47; accessed on 8 October 2026) was used only as a descriptive external reference with overall survival (OS) as the endpoint. Because treatment annotation in TCGA-DLBC is incomplete and survival endpoints in this cohort are limited by few events [23], it was not considered a validation cohort, and no assumption was made of uniform first-line R-CHOP treatment.

Baseline and post-infusion profiles from the single-arm ZUMA-1 study (GSE197977) [4,21] were excluded from the present analysis for four reasons. First, in preliminary comparisons between complete responders and non-complete responders, the direction of differential expression was strongly dependent on mean expression, and most associated transcripts showed expression levels close to the panel’s background noise; this pattern is consistent with technical variations (background noise, input RNA quantity, or normalization) that could not be resolved using the available processed data. Second, the baseline cohort was small (29 patients, 9 of whom did not achieve a complete response), and all patients in the paired post-infusion subgroup achieved a complete response, precluding inferences regarding resistance. Third, the absence of a randomized comparator group prevents any testing of treatment-specific effects. Fourth, differences in treatment line, panel content, outcomes, and response definitions limit comparability with the ZUMA-7 study. Therefore, all analyses, figures, and tables were restricted to ZUMA-7, using TCGA-DLBC as a descriptive reference. The derivation of the cohorts and the samples contributing to each analysis are summarized in Supplementary Figure S1.

### 2.2. Transcriptomic Data Acquisition and Processing

Normalized baseline expression data generated with the NanoString PanCancer IO 360 panel were retrieved from the Gene Expression Omnibus (GEO) under accession number GSE248835 (https://www.ncbi.nlm.nih.gov/geo/query/acc.cgi?acc=GSE248835; accessed on 8 October 2026; 256 samples; 817 features comprising individual mRNA transcripts and IO 360 signature scores). Only mRNA features were used for gene-level analyses; probes mapping to the same gene symbol were averaged. Values were log_2_-transformed (log_2_[x + 1]) only if they were not already on a logarithmic scale. The feature annotated as CD45RA in the panel corresponds to an isoform of *PTPRC*; because isoform-level quantification is not available in gene-level RNA-seq matrices, CD45RA was not evaluated in TCGA-DLBC.

For TCGA-DLBC, STAR-aligned RNA-seq expression (transcripts per million, TPM) and survival data were obtained from the GDC hub of UCSC Xena; Ensembl identifiers were mapped to gene symbols with org.Hs.eg.db, and OS time was converted from days to months. Because NanoString counts and RNA-seq TPM values are not directly comparable, no cross-platform merging or direct comparison of expression values was performed; all cross-cohort survival analyses relied on within-cohort median dichotomization.

### 2.3. Tumor Microenvironment Deconvolution

Immune and stromal populations were estimated with the Microenvironment Cell Populations-counter algorithm (MCP-counter; MCPcounter R package v1.2.0) [24]. Because MCP-counter was developed for genome-wide data, the presence of each population’s marker genes in the IO 360 panel was documented against the official marker list. Myeloid dendritic cells and fibroblasts had no available markers and could not be estimated; the remaining eight populations were compared between sustained response and treatment failure within each arm using the Wilcoxon rank-sum test, with Benjamini–Hochberg correction across the eight populations within each arm. No minimum coverage threshold was applied; populations with low coverage (e.g., neutrophils, 20%; monocytic lineage, 28.6%) are interpreted with caution.

### 2.4. Transcriptomic Mapping of Immune Evasion

Transcripts associated with T-cell exhaustion and immune checkpoints (*PDCD1*, *HAVCR2*, *LAG3*, *TIGIT*, *CTLA4*, *EOMES*, *ENTPD1*, *CXCL13*) and with the antigen-presentation machinery were investigated. The HLA complex was stratified a priori into four biological axes: classical class I molecules, the class I processing machinery, class II molecules, and non-classical class I molecules. Each gene was compared between response groups within each arm using the Wilcoxon rank-sum test and linear models adjusted for COO and SPD, with Benjamini–Hochberg correction applied separately within each gene panel and arm. For visualization, log_2_-scale values were scaled to Z-scores and displayed in heatmaps (ComplexHeatmap) [25], with hierarchical clustering (Euclidean distance, complete linkage) performed within each response stratum and annotated for response, clinical classification, COO, and SPD. Because clustering was performed within strata, the heatmaps are descriptive; inference rests on the per-gene tests.

### 2.5. Differential Expression and Pathway Enrichment

Within each arm, baseline differential expression between treatment failure and sustained response was assessed with limma [26] (moderated t-statistics; Benjamini–Hochberg-adjusted *p*-values reported). Because no transcript reached FDR < 0.05, an exploratory threshold (nominal *p* < 0.05 and |log_2_FC| > 0.5) was used only to prioritize candidates for survival screening. Gene set enrichment analysis (GSEA) of the MSigDB Hallmark collection [27] was performed with fgsea [28] on genes ranked by the limma moderated t-statistic (minimum and maximum gene set sizes 10 and 500). Because the contrast is failure versus sustained response, a positive normalized enrichment score (NES) indicates enrichment in treatment failure and a negative NES indicates enrichment in sustained response. For a descriptive per-patient view, single-sample pathway scores were obtained by running fgsea on each sample’s gene-centered expression ranking and averaging NES by response group within five functional categories; these scores were not used for inference.

### 2.6. Survival Screening and Multivariable Modeling

EFS (time in months and event indicator) was used as provided with the GEO dataset, as defined in the primary ZUMA-7 analysis [20]. All 25 axi-cel candidates and all 32 SOC candidates were screened within their own arm by (i) Kaplan–Meier estimates with the log-rank test after dichotomization at the intra-cohort median (high: expression ≥ median) and (ii) univariable Cox regression with expression modeled as a continuous log_2_ variable (survival and survminer R packages) [29,30]. Benjamini–Hochberg correction was applied across candidates within each arm and test. Hazard ratios (HR) therefore refer to a one-unit (log_2_) increase in expression, whereas Kaplan–Meier curves compare median-defined groups; the two analyses address different questions.

Candidates meeting a prespecified selection rule (log-rank *p* < 0.05 and univariable Cox *p* < 0.05) entered multivariable Cox models including the gene (continuous), SPD (continuous) and COO, fitted on complete cases after exclusion of patients with missing SPD (*n* = 121 patients and 69 events in the axi-cel arm; 13 patients lacked SPD). COO was entered as a four-level categorical covariate as annotated in GEO (axi-cel complete cases: GCB, *n* = 89; ABC, *n* = 12; unclassified, *n* = 15; “null” [not reported], *n* = 5); no patient was coded as “unknown”, so no patient was excluded for COO, and “null” was retained as a separate category rather than recoded as missing. Excluding the five patients with “null” COO did not change the direction or nominal significance of any of the 13 multivariable associations (*n* = 116). Benjamini–Hochberg correction was applied across all 14 modeled genes, of which 13 showed multivariable *p* < 0.05. The complete analysis included RORC. Because candidate selection and modeling used the same outcome in the same patients, multivariable *p*-values are conditional on selection and are not interpreted as validation. The proportional hazards assumption was assessed with scaled Schoenfeld residuals (cox.zph; per-covariate and global tests) for every multivariable and interaction model, and median follow-up was estimated with the reverse Kaplan–Meier method. To assess the dependence of the screening FDR on the prioritization step, the log-rank and univariable Cox tests were repeated across all 769 mRNA features of the IO 360 panel in the axi-cel arm, with Benjamini–Hochberg correction across the whole panel. As a crude proxy for tumor content, the multivariable models of selected transcripts were refitted with the MCP-counter B-lineage score or the IO 360 B-cell signature score as an additional covariate.

### 2.7. Assessment of Treatment Specificity

Predictive (treatment-specific) value was assessed formally with gene-by-treatment interaction models in the pooled randomized ZUMA-7 population. For each candidate in the union of the arm-specific candidate sets (25 axi-cel and 32 SOC candidates; *KLRD1* was present in both lists, giving 56 unique genes), a Cox model for EFS included the gene (continuous log_2_), treatment arm (SOC as reference), their interaction, SPD, and COO. The interaction HR is the ratio of the per-log_2_ HR in the axi-cel arm to that in the SOC arm; Benjamini–Hochberg correction was applied across all tested genes. As a descriptive complement, Kaplan–Meier analyses of the multivariable candidates and of interaction-derived candidates were repeated in the axi-cel arm, the SOC arm, and TCGA-DLBC (OS), with Benjamini–Hochberg correction within each gene set and cohort. A difference in statistical significance between cohorts was not considered evidence of a differential effect; only the interaction tests were used to address treatment specificity. In total, 56 genes were tested (228 patients with complete covariates; 161 events). Because candidates were prioritized on their association with outcome within one arm, the interaction estimates are subject to the same selection and are expected to be biased away from the null; interaction results, including those with FDR < 0.05, are therefore hypothesis-generating and require confirmation in independent randomized data. Because the treatment-arm term violated the proportional hazards assumption, the interaction models were refitted as sensitivity analyses (i) stratified by treatment arm (strata[arm], with gene, gene × arm, SPD and COO), which removes the proportional-hazards requirement for the arm main effect; (ii) stratified by both arm and COO; and (iii) with a time-split interaction in which follow-up was split at 6 months and period-specific gene × arm terms were estimated in an arm-stratified model with robust (patient-clustered) standard errors. In the pooled complete cases (*n* = 228), COO was GCB in 172, ABC in 20, unclassified in 26, and “null” in 10 patients.

### 2.8. Literature Review Strategy for Prognostic Candidates

For the transcripts retained in the multivariable model, published evidence was reviewed, prioritizing studies in DLBCL and other B-cell lymphomas, particularly those involving anti-CD19 CAR T-cell therapy; evidence from other tumor types or normal immune cells was considered only when lymphoma-specific data were not available. Because the present analyses reuse the ZUMA-7 dataset, agreement with the primary ZUMA-7 report [20] was interpreted as concordance rather than independent validation.

### 2.9. Integrated Conceptual Model

To summarize the findings, a conceptual model was built from (I) the direction of Hallmark pathway enrichment (GSEA), (II) the multivariable Cox estimates in the axi-cel arm, and (III) the treatment-by-gene interaction results. The diagram, dichotomized into states associated with treatment failure and with sensitivity, is a hypothesis-generating summary of transcriptional associations and does not represent demonstrated causal mechanisms. It was created with BioRender (Created in BioRender. Figueiredo, B. (2026) https://BioRender.com/ifdik9o).

### 2.10. Statistical Analysis and Data Visualization

All analyses were performed in R (version 4.6.1). Two-group comparisons used the Wilcoxon rank-sum (Mann–Whitney U) test [31,32]. Survival was analyzed with Kaplan–Meier estimates, the log-rank test, and Cox proportional hazards regression, with HRs and 95% confidence intervals (CIs). Multiplicity was controlled with the Benjamini–Hochberg false discovery rate (FDR) [33], applied within the following families: (i) each gene panel and arm for the B-cell, exhaustion and HLA analyses; (ii) all genes in each limma contrast; (iii) all Hallmark sets in each GSEA; (iv) all candidates in each arm-specific survival screen; (v) all modeled genes in the multivariable analysis; (vi) all genes tested for interaction; (vii) each gene set and cohort in the descriptive cross-context analyses, which, because these genes had already been selected based on the same outcome, are reported for descriptive purposes only and do not replace the FDR-adjusted values obtained during the initial screening; (viii) the eight MCP-counter populations within each arm; (ix) all 769 mRNA features in the panel-wide screen; and (x) all 56 genes within each interaction sensitivity model. Tests were two-sided with α = 0.05; results with FDR < 0.05 are described as FDR-supported and nominal results as exploratory. Differential expression, enrichment, and survival analyses used limma (v3.68.4) [26], fgsea (v1.38.0) [28], and survival (v3.8-12) [29]. Visualizations used ggplot2 (v4.0.3) [34], ComplexHeatmap (v2.28.0) [25], and survminer (v0.5.2) [30]. All scripts are available at https://github.com/jeanssresende/DLBCL-CD19-Analysis (accessed on 8 October 2026).

## 3. Results

### 3.1. Baseline B-Cell Lineage and Differentiation Transcripts Are Associated with Sustained Response to Axi-cel

**Figure 1:**
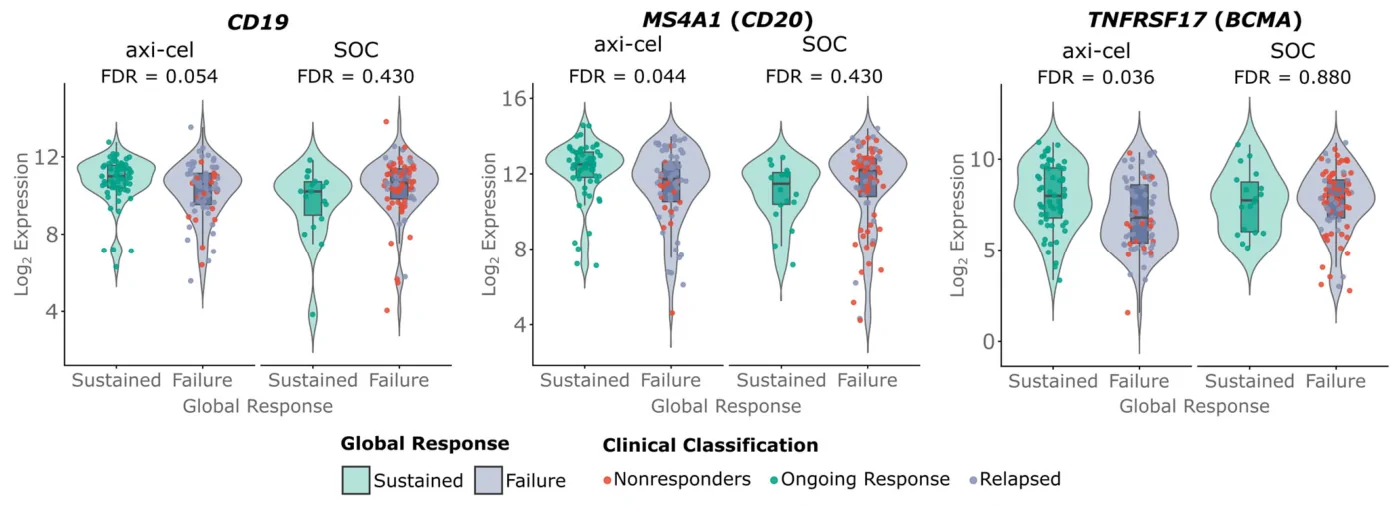
Baseline expression of key B-cell lineage and differentiation markers from the NanoString IO360 panel and their association with clinical outcomes in the ZUMA-7 study. Pre-infusion (baseline) mRNA expression levels (log_2_) of the therapeutic target CD19, along with *MS4A1* and *TNFRSF17* (which demonstrated significant FDR control in the Wilcoxon test), were assessed in the axi-cel and SOC treatment arms. Patients are stratified according to durable clinical outcome (sustained response versus failure). Comparisons between groups were performed using the Wilcoxon rank-sum test, applying Benjamini–Hochberg FDR corrections (detailed linear model results, adjusted for COO and baseline SPD, are presented in Supplementary Table S1). Box plots overlaid on violin plots indicate the median and interquartile range, with individual data points color-coded according to detailed clinical classifications.

Clinical assessment of the therapeutic target is usually limited to CD19 itself. We therefore examined *CD19* together with other genes of the IO 360 B-cell signature to explore whether the broader B-cell transcriptional program is associated with axi-cel outcome. Within the axi-cel cohort of the ZUMA-7 study, patients who achieved a sustained clinical response exhibited a consistent pattern toward higher baseline expression across the evaluated panel of canonical B-cell lineage markers (*CD19*, *MS4A1*) and the late-differentiation state marker (*TNFRSF17*/*BCMA*) compared to those who experienced therapeutic failure (Figure 1). Specifically, while *CD19* displayed a trending association, *MS4A1* and *TNFRSF17* reached FDR < 0.05 (Wilcoxon test FDR = 0.054, 0.044, and 0.036, respectively; Supplementary Table S1). In linear models adjusted for COO and SPD, the associations remained FDR-supported for *MS4A1* and *TNFRSF17* (FDR = 0.032 for both) but not for *CD19* (FDR = 0.192; Supplementary Table S1). *TNFRSF17* (BCMA), which is characteristically expressed at late B-cell/plasma-cell differentiation stages and is part of the IO 360 B-cell signature, is interpreted here as a differentiation-state marker rather than a canonical lineage marker. This profile is consistent with the hypothesis that CAR T-cell sensitivity is associated with preservation of a mature, differentiated B-cell transcriptional program in the tumor, of which CD19 is one component.

In the SOC arm, none of the B-cell signature genes differed between response groups after FDR correction (all Wilcoxon FDR ≥ 0.430; all adjusted-model FDR ≥ 0.276; Supplementary Table S1). This contrast between arms is descriptive and does not by itself establish that the association is specific to cellular immunotherapy; treatment specificity was assessed formally with interaction models (Section 3.4). Taken together, these findings are consistent with the primary ZUMA-7 analysis, in which B-cell-related features were associated with favorable outcomes after axi-cel [20].

### 3.2. Baseline T-Cell Exhaustion and HLA Transcripts Were Not Associated with Response in Either ZUMA-7 Arm

**Figure 2:**
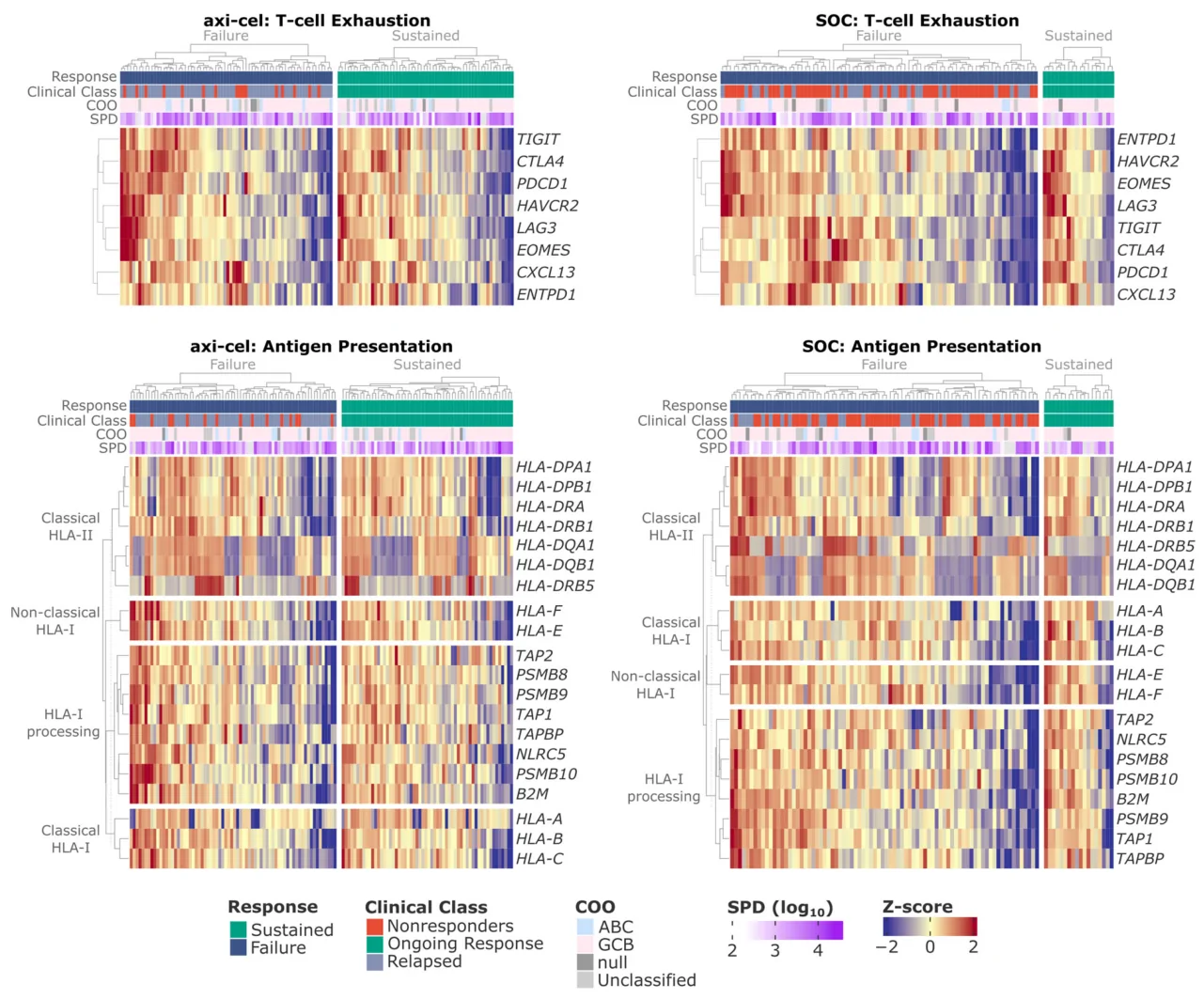
Transcriptional profile of immune evasion mechanisms: T-cell exhaustion and regulation of the antigen presentation machinery (HLA) in the ZUMA-7 study cohort. Normalized gene expression profiles (Z-score) mapping the architecture of the immune microenvironment in the axi-cel and standard of care (SOC) treatment arms. The upper heatmaps display the expression of genes characteristic of T-cell exhaustion and immune checkpoints (*TIGIT*, *CTLA4*, *PDCD1*, *HAVCR2*, *LAG3*, *EOMES*, *CXCL13*, *ENTPD1*), with comprehensive statistical assessments detailed in Supplementary Table S2. The lower heatmaps illustrate the functional integrity of the antigen presentation machinery, biologically subdivided into canonical Class II molecules, non-classical Class I molecules, intracellular processing and transport complexes, and classical Class I molecules, with statistical metrics provided in Supplementary Table S3. Patients (columns) are hierarchically clustered (Euclidean distance, complete linkage) within each clinical response stratum (sustained response versus failure); because clustering is performed within strata, the display is descriptive and does not test segregation by outcome. Top annotations provide individualized clinical context for each sample: overall response, detailed clinical classification, molecular/cell-of-origin (COO) subgroup, and baseline tumor burden mapped on a continuous logarithmic scale (SPD). Expression divergence is mapped on a Z-score scale, where intense blue represents underexpression (−2) and dark red represents overexpression (+2) relative to the cohort mean.

In classical tumor immunology, immune evasion is often attributed to T-cell exhaustion within the microenvironment or by the suppression of the human leukocyte antigen (HLA) presentation machinery. However, our analyses did not identify these canonical axes as determinants of therapeutic failure with axi-cel. Quantitative assessment of canonical T-cell exhaustion and immune checkpoint signatures (*TIGIT*, *CTLA4*, *PDCD1*, *HAVCR2*, *LAG3*, *EOMES*, *CXCL13*, *ENTPD1*) (Figure 2) revealed no differential expression between patients with sustained responses and those who experienced therapeutic failure after FDR correction (axi-cel arm: all FDR values by Wilcoxon test = 0.987 and all FDR values in adjusted models ≥ 0.541; SOC arm: all FDR values ≥ 0.613 for the Wilcoxon test and all FDR values in adjusted models = 0.829; Supplementary Table S2). As demonstrated by continuous statistical tests and linear models adjusted for COO and SPD, this pattern of non-discrimination was consistent in both ZUMA-7 arms.

Similarly, the functional and structural assessment of HLA complex integrity demonstrated intra-cohort heterogeneity. Because samples in Figure 2 were clustered within each response stratum, the heatmaps are descriptive and cannot test whether clusters segregate by outcome; the inference rests on the per-gene tests, in which no classical class I, processing, class II, or non-classical HLA transcript differed between response groups after FDR correction (axi-cel arm: all Wilcoxon FDR ≥ 0.854; SOC arm: all Wilcoxon FDR ≥ 0.615; Supplementary Table S3). Given the group sizes (58 vs. 70 patients in the axi-cel arm and 18 vs. 80 in the SOC arm), moderate associations cannot be excluded.

Because CAR recognition is HLA-independent, these data are biologically plausible and do not support pre-existing T-cell exhaustion or HLA loss, as captured by bulk baseline transcripts, as major determinants of axi-cel outcome; other features of tumor state were therefore investigated.

### 3.3. Exploratory Differential Expression and Pathway Enrichment

To decipher resistance mechanisms extending beyond classic immune escape, we performed differential gene expression analysis integrated with pathway enrichment analysis (GSEA, with descriptive per-sample enrichment scores). Acknowledging the limitations inherent in multiple testing during transcriptomic profiling, this initial discovery step was explicitly defined as a hypothesis-generating phase.

Using an exploratory threshold (nominal *p* < 0.05 and |log_2_FC| > 0.5), 25 transcripts were prioritized in the axi-cel arm (Supplementary Table S4) and 32 in the SOC arm (Supplementary Table S5). In the axi-cel arm, transcripts with higher expression in treatment failure included *MET*, *KLRD1*, *DUSP5*, *WNT11*, *CDH2* and *NCAM1*, whereas *TNFRSF17*, *MS4A1*, *IRF8*, *BTLA*, *HLA-DOB*, *CD45RA* and *CD19* were higher in sustained responders. No transcript reached FDR < 0.05 (minimum adjusted *p* = 0.600 in the axi-cel arm and 0.800 in the SOC arm); this layer is therefore hypothesis-generating and served only to prioritize candidates for survival screening.

**Figure 3:**
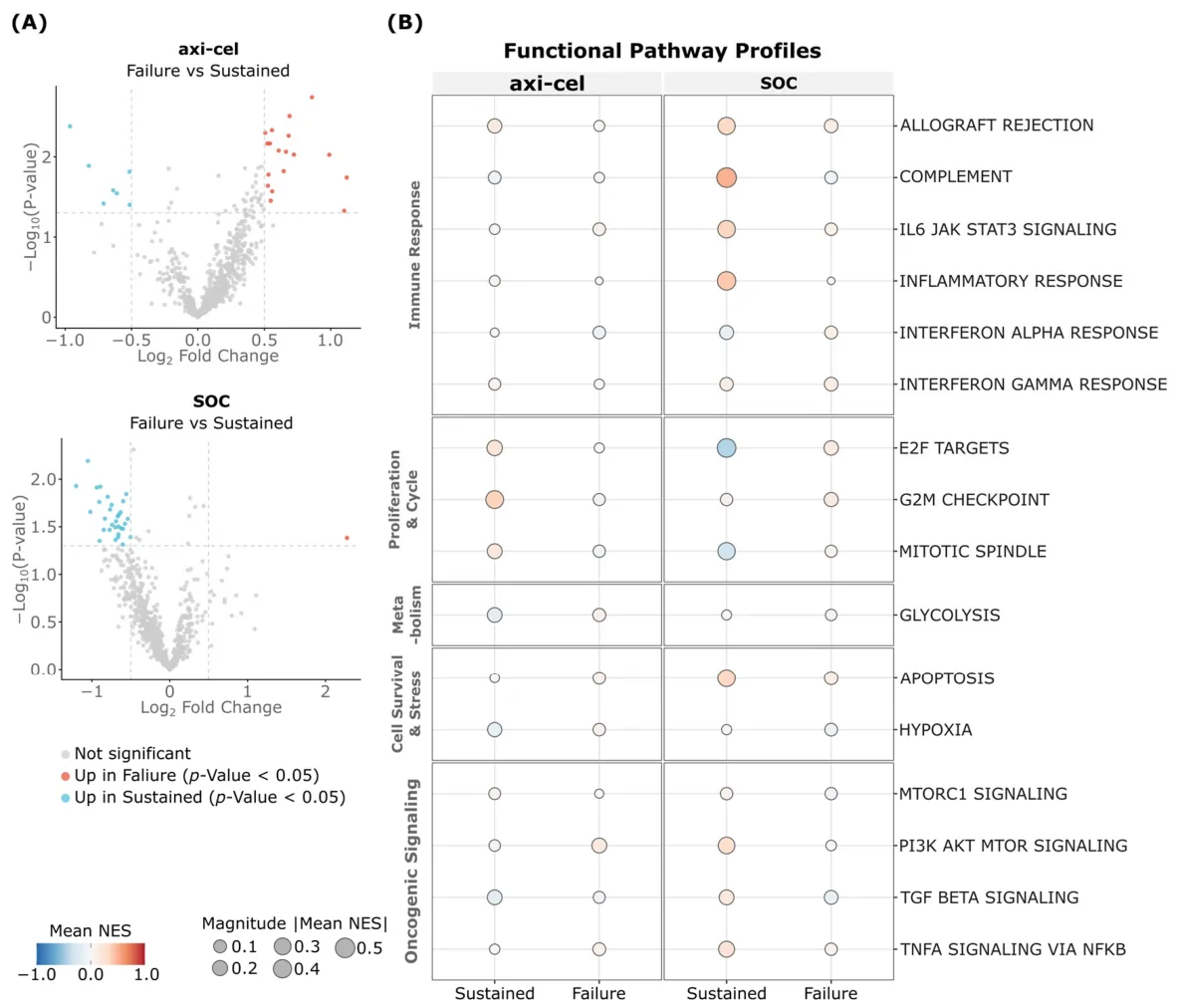
Exploratory differential expression and pathway enrichment by response group in the ZUMA-7 arms. (**A**) Volcano plots showing differential gene expression analysis (DEA) of the NanoString IO360 panel prior to infusion, comparing clinical response groups (Failure vs. Sustained) in the axi-cel (top) and standard of care/SOC (bottom) arms. Significance is highlighted based on an exploratory threshold of nominal (unadjusted) *p*-value < 0.05 and absolute log_2_ fold change > 0.5 (dashed lines; detailed metrics are provided in Supplementary Tables S4 and S5). (**B**) Descriptive single-sample pathway profiles (per-sample GSEA on gene-centered expression). MSigDB Hallmark gene sets were grouped into five functional categories (y-axis). Circle color indicates the Mean Normalized Enrichment Score (Mean NES), reflecting relative enrichment (red) or depletion (blue) of panel genes from each set in each clinical group. Circle size is proportional to the absolute mean NES. Full GSEA results for the axi-cel and SOC arms are detailed in Supplementary Tables S6 and S7, respectively.

In the axi-cel arm, GSEA showed a trend toward enrichment of Notch signaling in treatment failure (NES = 1.74; FDR = 0.055), whereas E2F targets (NES = −1.81; FDR = 0.055) and the G2/M checkpoint (NES = −1.94; FDR = 0.055) showed a trend toward enrichment in patients with a sustained response (Figure 3; Supplementary Table S6); no Hallmark set reached FDR < 0.05. The Hallmark epithelial–mesenchymal transition set showed only a nominal trend toward enrichment in treatment failure (NES = 1.43; *p* = 0.041; FDR = 0.194; Supplementary Table S6). In the SOC arm, by contrast, E2F targets (NES = 3.16; FDR < 0.001) and the mitotic spindle (NES = 2.55; FDR = 0.001) were enriched in patients with treatment failure (Supplementary Table S7). The single-sample pathway scores (Figure 3B) are shown descriptively.

### 3.4. Survival Screening, Multivariable Modeling and Treatment-by-Gene Interaction

The 25 axi-cel candidates were screened for association with EFS (134 patients; 78 events; median follow-up by reverse Kaplan–Meier, 23.7 months [IQR 19.5–24.5]; 21.6 months [IQR 18.3–26.8] in the SOC arm) by the log-rank test and univariable Cox regression (Supplementary Table S8; parallel results for the 32 SOC candidates are shown in Supplementary Table S9). After Benjamini–Hochberg correction across the 25 candidates, nine transcripts were FDR-supported by both tests within this prioritized family: *DUSP5*, *MS4A1*, *CD45RA*, *KLRD1*, *WNT11*, *CD19*, *HLA-DOB*, *TNFRSF17* and *RORC*. Fourteen transcripts met the prespecified selection rule (log-rank *p* < 0.05 and univariable Cox *p* < 0.05) and entered multivariable models.

**Figure 4:**
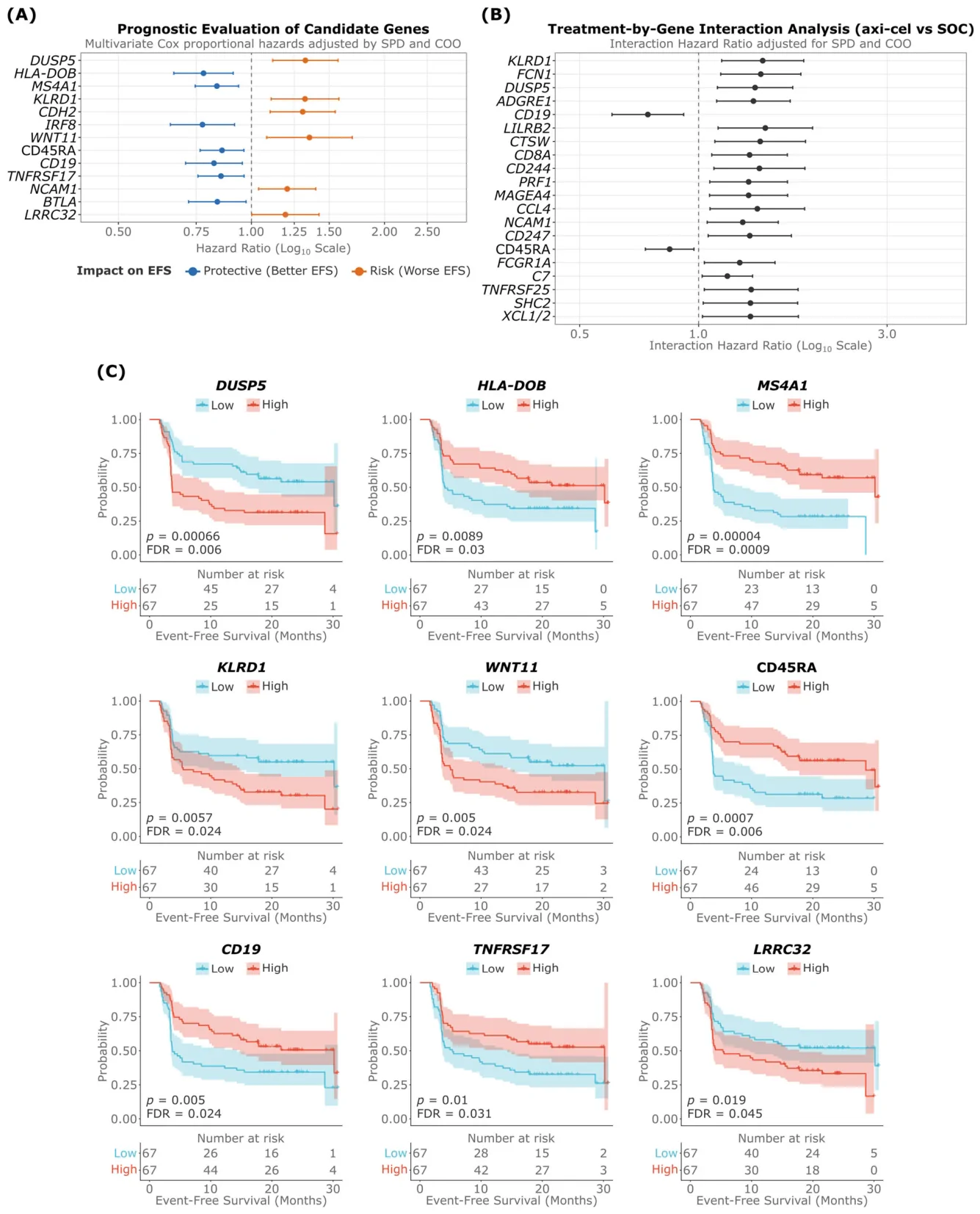
Prognostic evaluation of nominally prioritized candidate genes and treatment-gene interaction analyses in the ZUMA-7 study cohort. (**A**) Forest plot of the multivariable Cox proportional hazards model, evaluating the prognostic association of candidate genes with event-free survival (EFS) in the axi-cel-treated arm (HR per log_2_ unit; *n* = 121; 69 events), adjusted for baseline tumor burden (SPD) and lymphoma cell-of-origin (COO) subtype (metrics detailed in Supplementary Table S10). Hazard ratios greater than 1 (orange) indicate markers associated with a higher risk of treatment failure/worse EFS, whereas values less than 1 (blue) indicate an association with longer EFS. (**B**) Forest plot of the treatment-gene interaction, showing the 20 candidates with the smallest interaction *p*-values in the combined ZUMA-7 population (FDR < 0.1); the interaction HR is the ratio of the per-log_2_ HR in the axi-cel arm to that in the SOC arm (reference), adjusted for SPD and COO (complete metrics in Supplementary Table S11). (**C**) Representative Kaplan–Meier survival curves for EFS, illustrating patient stratification based on the median expression of candidate genes (High in red versus Low in blue) in the axi-cel cohort for candidate genes with a Kaplan–Meier FDR < 0.05, including 95% confidence intervals, log-rank test *p*-values, FDR adjustments across the 25 axi-cel candidates (Supplementary Table S8), and numbers-at-risk tables.

In multivariable models adjusted for SPD and COO (*n* = 121; 69 events), 13 of the 14 transcripts remained associated with EFS (Figure 4A; Supplementary Table S10); HRs are expressed per log_2_ unit. Protective associations (HR < 1) were observed for *MS4A1* (HR 0.835; 95% CI 0.75–0.94), *CD19* (0.823; 0.71–0.95), *TNFRSF17* (0.853; 0.76–0.96), *IRF8* (0.775; 0.66–0.92), *HLA-DOB* (0.779; 0.67–0.91), *BTLA* (0.837; 0.72–0.97) and *CD45RA* (0.858; 0.77–0.96), and adverse associations (HR > 1) for *DUSP5* (1.324; 1.12–1.57), *KLRD1* (1.322; 1.11–1.58), *CDH2* (1.306; 1.10–1.55), *WNT11* (1.353; 1.08–1.69), *NCAM1* (1.205; 1.04–1.40) and *LRRC32* (1.194; 1.00–1.42; *p* = 0.048; FDR = 0.051). *RORC* did not remain significant (HR 1.181; 95% CI 0.977–1.428; *p* = 0.086; Supplementary Table S17). Eight transcripts (*MS4A1*, *CD19*, *TNFRSF17*, *HLA-DOB*, *CD45RA*, *DUSP5*, *KLRD1* and *WNT11*) were FDR-supported in the log-rank, univariable and multivariable analyses within the 25 prioritized candidates and form a core candidate set; *IRF8*, *BTLA*, *CDH2* and *NCAM1* (log-rank FDR = 0.062–0.068) and *LRRC32* (univariable FDR = 0.055) did not meet FDR < 0.05 at every stage and are considered exploratory. Because selection and modeling used the same outcome in the same patients, these estimates are conditional on selection and may be inflated. Because the 25 candidates were prefiltered for nominal differential expression between response groups, an outcome correlated with EFS, this FDR family is enriched for EFS associations. In a panel-wide screen of all 769 mRNA features in the axi-cel arm, no transcript was FDR-supported by both tests; among the eight core genes, only *MS4A1* remained FDR-supported in the log-rank test (FDR = 0.029), and the lowest panel-wide univariable Cox FDR was 0.174 (*DUSP5*) (Supplementary Table S20). The core set should therefore be read as FDR-supported only within the prioritized candidates. With median dichotomization, nine of the 13 multivariable candidates stratified EFS with KM FDR < 0.05 (Figure 4C). Scaled Schoenfeld residuals showed no evidence of non-proportional hazards for 12 of the 13 models (global *p* ≥ 0.083); for *CDH2*, the gene term (*p* = 0.038) and the global test (*p* = 0.013) suggested non-proportionality, so its HR should be read as an average over follow-up (Supplementary Table S17). When the MCP-counter B-lineage score or the IO 360 B-cell signature was added as a crude proxy for tumor content, the associations of *CDH2* (HR 1.279, *p* = 0.006; and 1.304, *p* = 0.004) and *DUSP5* (1.275, *p* = 0.007; and 1.256, *p* = 0.012) persisted, whereas those of *NCAM1*, *WNT11* and *KLRD1* were attenuated and no longer nominally significant (*p* = 0.057–0.155), and *LRRC32* gave inconsistent results (*p* = 0.027 and 0.068) (Supplementary Table S21).

In the pooled randomized population (Figure 4B; Supplementary Table S11), 22 of the 56 tested candidates showed a nominal gene-by-treatment interaction (*p* < 0.05), but only four reached FDR < 0.05: *KLRD1* (interaction HR 1.45; 95% CI 1.14–1.85), *FCN1* (1.44; 1.14–1.81), *DUSP5* (1.39; 1.12–1.73) and *ADGRE1* (1.38; 1.11–1.71; FDR = 0.049 for all four). *CD19* showed a borderline interaction (0.74; 0.60–0.92; *p* = 0.006; FDR = 0.053), and *NCAM1* (1.29; 1.05–1.59; FDR = 0.064) and *CD45RA* (0.84; 0.73–0.97; FDR = 0.073) nominal interactions. The remaining eight transcripts of the multivariable panel (*MS4A1*, *TNFRSF17*, *IRF8*, *HLA-DOB*, *BTLA*, *CDH2*, *WNT11,* and *LRRC32*) did not show nominal evidence of interaction and should be regarded as prognostic within the axi-cel arm, without evidence of treatment specificity. *FCN1* and *ADGRE1*, both myeloid-associated, originated from the SOC screen, in which higher expression was associated with a lower hazard (univariable HR 0.80 and 0.85, respectively; Supplementary Table S9). Because candidates were prioritized on outcome within one arm, these interaction estimates are subject to selection and are hypothesis-generating. Moreover, the treatment-arm and interaction terms violated the proportional hazards assumption in all interaction models (Schoenfeld *p* < 0.001; Supplementary Table S17), consistent with the time-varying separation of EFS curves between arms, so interaction HRs represent averages over follow-up. In sensitivity analyses stratified by treatment arm, the interaction estimates were essentially unchanged: *KLRD1*, HR 1.47 (95% CI 1.15–1.86; *p* = 0.002; FDR = 0.047) and *DUSP5*, HR 1.43 (1.15–1.78; *p* = 0.001; FDR = 0.047), with no evidence of non-proportionality for the interaction terms (Schoenfeld *p* = 0.22 and 0.73). The residual non-proportionality in these models was attributable to COO; additional stratification by COO gave similar estimates (*KLRD1*, 1.47 [1.16–1.87]; *DUSP5*, 1.50 [1.20–1.89]; global Schoenfeld *p* = 0.46 and 0.98), although more genes reached FDR < 0.05 under this specification (Supplementary Table S18). In time-split models, the interaction HRs exceeded 1 both before 6 months (*KLRD1*, 1.33 [1.04–1.71]; *DUSP5*, 1.36 [1.07–1.74]; 128 events) and after 6 months (2.18 [1.31–3.62] and 1.82 [1.02–3.23]; 33 events) (Supplementary Table S19; Supplementary Figure S2). These analyses indicate robustness to the proportional-hazards violation but do not remove the selection bias described above, and the interactions are still described as borderline.

### 3.5. Descriptive Cross-Context Comparison (ZUMA-7 SOC Arm and TCGA-DLBC)

As a descriptive complement to the interaction tests, the candidate transcripts derived from the multivariable model (Figure 4A; Supplementary Table S12) and from the interaction analysis (Figure 4B; Supplementary Table S13) were examined by Kaplan–Meier analysis in the axi-cel and SOC arms of ZUMA-7 and in TCGA-DLBC, which served only as an external descriptive reference with incomplete treatment annotation and few events. Differences in significance between settings were not interpreted as evidence of treatment specificity.

**Figure 5:**
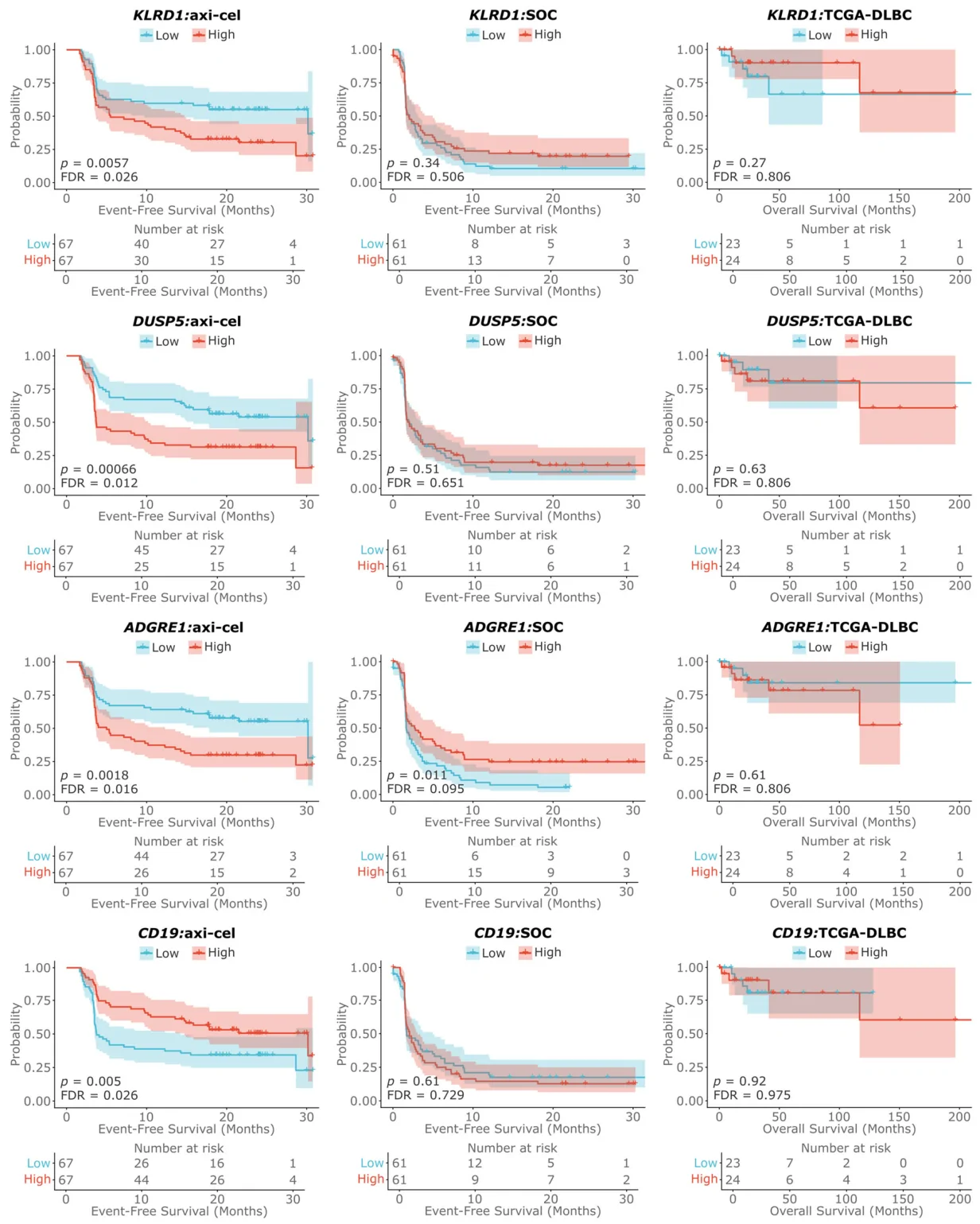
Descriptive comparison of interaction-derived candidate genes across clinical settings of large B-cell lymphoma. Kaplan–Meier curves describing baseline expression of candidate genes from the treatment-by-gene interaction analysis (nominal interaction *p* < 0.05; Supplementary Table S13), restricted to those with KM FDR < 0.05 in the axi-cel arm (KLRD1, DUSP5, ADGRE1 and CD19), in three settings: (1) ZUMA-7 axi-cel: Event-Free Survival (EFS) in patients treated with anti-CD19 CAR T-cell therapy; (2) ZUMA-7 SOC: EFS in the randomized control arm treated with standard salvage chemotherapy; and (3) TCGA-DLBC: Overall Survival (OS) in an external exploratory cohort with incomplete treatment annotation (*n* = 47; 9 deaths). Patients were stratified into high-expression (High, red line) and low-expression (Low, blue line) groups based on the intra-cohort median expression threshold for each transcript. Follow-up time (x-axis) is consistently presented in months across all cohorts. Statistical significance was determined using the log-rank test, including 95% confidence intervals, FDR adjustments across the interaction-derived genes within each cohort, and numbers-at-risk tables.

All 12 multivariable candidates evaluable in the three settings were nominally associated with EFS in the axi-cel arm (log-rank *p* ≤ 0.043; Supplementary Table S12). Because these genes were selected on the same outcome in the same patients, this is a restatement of the selection rather than additional evidence, and the FDR values over the 25 screened candidates (Supplementary Table S8) remain the relevant ones. None of these genes was associated with EFS in the SOC arm (all *p* ≥ 0.133; FDR ≥ 0.730), and in TCGA-DLBC (47 patients; 9 deaths) only *WNT11* reached nominal significance (*p* = 0.036; FDR = 0.431) (Supplementary Table S12). Among the interaction-derived candidates (Supplementary Table S13), four reached KM FDR < 0.05 in the axi-cel arm (*DUSP5*, FDR = 0.012; *ADGRE1*, 0.016; *KLRD1*, 0.026; *CD19*, 0.026; Figure 5). *ADGRE1* also stratified EFS in the SOC arm, in the opposite direction (*p* = 0.011; FDR = 0.095), and *SHC2* stratified EFS only in the SOC arm (*p* = 0.002; FDR = 0.039). These descriptive contrasts are interpreted together with, and not in place of, the formal interaction tests; with only nine events, TCGA-DLBC is underpowered, and absence of significance in this cohort is not evidence of absence of an effect.

### 3.6. Microenvironmental Deconvolution and Cellular Composition Analysis

To assess whether bulk estimates of immune and stromal populations were associated with response, we conducted an exploratory assessment of the overall microenvironment composition using the MCP-counter deconvolution method. Given that our transcriptomic profiling relied on the NanoString IO360 targeted panel rather than whole-transcriptome sequencing, we first evaluated the presence of canonical MCP-counter marker genes within our dataset (Supplementary Table S14). Although certain major lineages, such as CD8 T cells, showed complete coverage (*CD8B*, albeit represented by a single marker gene) and compartments such as T cells, cytotoxic lymphocytes, and B lineage cells demonstrated partial representation (ranging from 44.4% to 57.1% based on available markers like *CD3D*, *CD3G*, *CD28*, *CD8A*, *CD19*, and *MS4A1*), other compartments exhibited limited coverage (e.g., monocytic lineage and endothelial cells) or a complete absence of algorithm-specific transcripts (such as fibroblasts and myeloid dendritic cells) (Supplementary Table S14).

Myeloid dendritic cells and fibroblasts could not be estimated, and neutrophils (20%) and the monocytic lineage (28.6%) had low marker coverage (Supplementary Table S14). In the axi-cel arm, only the B-lineage score differed between response groups after FDR correction (median 11.37 in sustained response vs. 10.95 in treatment failure; *p* = 0.004; FDR = 0.029; Supplementary Table S15). Because this score is derived from *CD19*, *CD79A*, *FCRL2,* and *MS4A1*, it largely reflects malignant B-cell content and is not an independent microenvironmental variable. No other immune or stromal population stratified response after FDR correction. In the SOC arm, no population reached FDR < 0.05 (all FDR ≥ 0.115); the B-lineage score was nominally higher in treatment failure (median 10.98 vs. 10.17; *p* = 0.029; FDR = 0.115; Supplementary Table S16). Within the limits of panel coverage, these results suggest that gene-level transcriptional features are more informative than bulk estimates of immune or stromal populations.

### 3.7. Literature-Based Interpretation of Prognostic Candidates

To contextualize the 13 transcripts that remained associated with EFS after adjustment for SPD and COO in the axi-cel arm (12 with FDR < 0.05 and *LRRC32* at FDR = 0.051; Figure 4A; Supplementary Table S10), we reviewed the published evidence available for each marker. Priority was given to studies in DLBCL and other B-cell lymphomas, particularly those involving anti-CD19 CAR T-cell therapy; evidence from other tumor types or from normal immune cells was considered only when lymphoma-specific data were not available. Because the present analyses reuse the ZUMA-7 dataset, agreement with the primary ZUMA-7 report [20] is interpreted as concordance rather than independent validation. The results are summarized in Table 1.

Among the protective markers, the strongest external support came from studies in DLBCL and LBCL. CD19 and MS4A1 are directly linked to antigen availability and B-cell identity, whose loss or reduction has been associated with failure of CAR T-cell therapy and of chemoimmunotherapy, respectively [16,17]. *IRF8* and *HLA-DOB* converge on B-cell differentiation and MHC class II antigen processing, pathways whose disruption has been implicated in immune evasion and adverse outcomes in DLBCL [19,38]. Notably, *IRF8* and *BTLA* have been reported with opposite prognostic directions depending on the treatment setting or on the cellular compartment in which they are expressed [36,41,42], reinforcing that bulk transcript levels should be interpreted in light of the therapeutic context and the cell type of origin.

In contrast, evidence for the adverse markers is scarcer in DLBCL. *NCAM1* (CD56) and *WNT11* have been described in DLBCL subsets with aggressive clinical or molecular features [51,54], whereas *CDH2*, *DUSP5*, and *LRRC32* have been characterized mainly in other tumor types or in regulatory immune compartments, where they are linked to EMT-like plasticity and adhesion-mediated therapy resistance, altered MAPK signaling, and TGF-β-dependent immunosuppression, respectively [46,50,55]. *KLRD1* illustrates how a single marker may carry distinct meanings, since NK-like reprogramming has been associated both with CAR T-cell dysfunction and, in LBCL, with remission [48,49]. These adverse genes should therefore be regarded as new candidate correlates of axi-cel failure in LBCL rather than replications of established markers, and their cellular source and functional relevance require evaluation in independent cohorts and mechanistic studies.

### 3.8. Integrated Model of Transcriptional States Associated with CAR T-Cell Response

**Figure 6:**
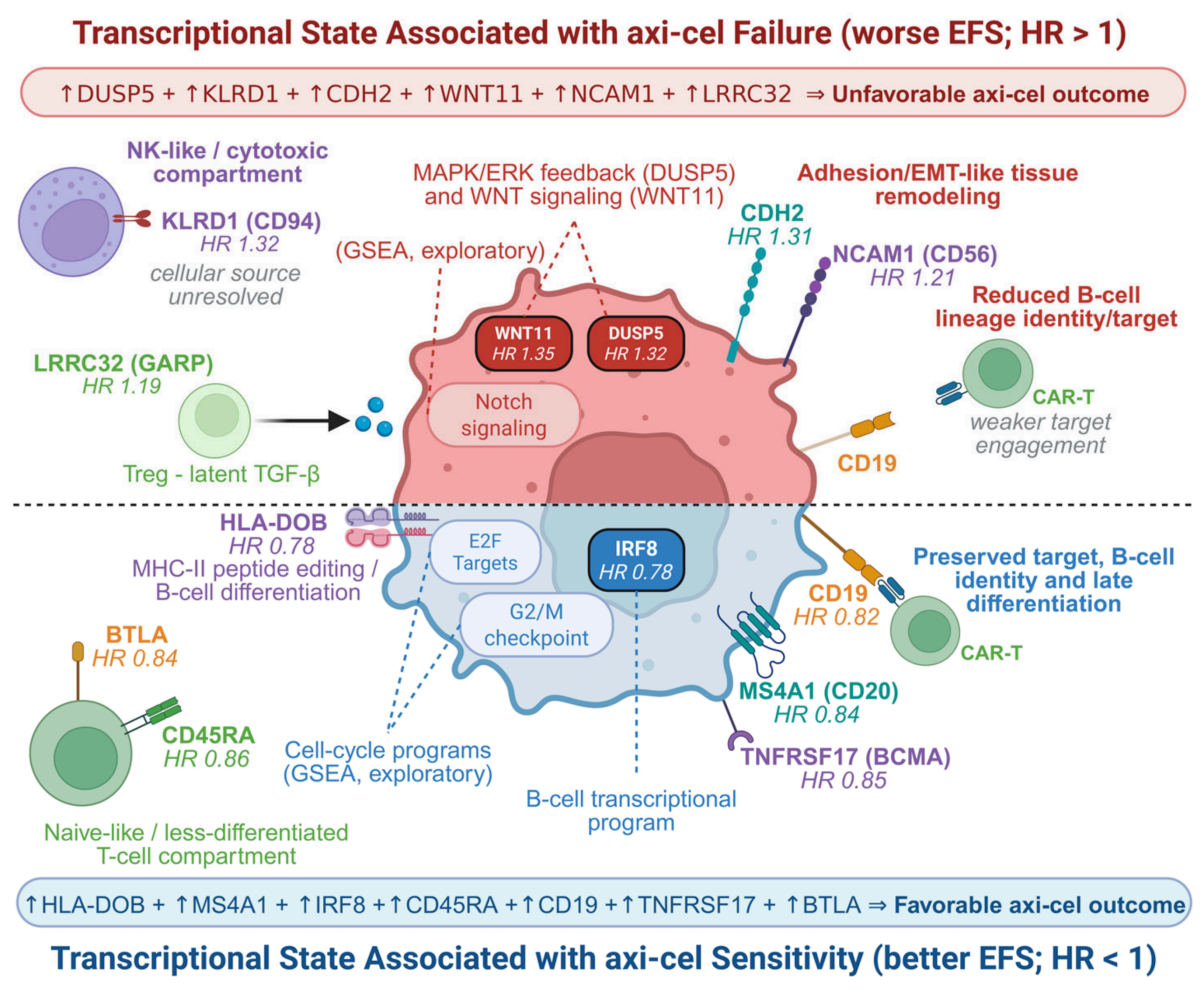
Integrated conceptual model of transcriptional states associated with therapeutic sensitivity and resistance to axi-cel. The model summarizes tumor-intrinsic and microenvironmental signatures identified through cross-sectional analyses of the ZUMA-7 study dataset. (**Top panel**, in red): The state associated with treatment failure is characterized by lower expression of B-cell transcripts, accompanied by adverse transcriptional features, including *DUSP5*, *WNT11*, *CDH2*, *NCAM1*, *LRRC32,* and *KLRD1*-tentatively interpreted as altered adhesion, developmental signaling, and NK/cytotoxic-compartment features; the EMT-like label interprets individual exploratory transcripts and is not supported by pathway-level enrichment at FDR < 0.05. Lower B-cell transcript levels may also reflect a lower tumor-cell fraction of the biopsy. (**Bottom panel**, in blue): The therapeutic sensitivity state is marked by higher expression of mature B-cell transcripts (*CD19*, *MS4A1*, *TNFRSF17*, *IRF8*) and late differentiation states, alongside favorable associations with *HLA-DOB*, *BTLA*, and the CD45RA isoform (*PTPRC*). Arrows represent proposed biological relationships derived from transcriptional associations, rather than direct causal mechanisms. Abbreviations: EMT-like, epithelial–mesenchymal transition-like program; HR, hazard ratio for event-free survival.

To integrate the transcriptomic and clinical associations identified in this study, we developed a conceptual model summarizing the biological states associated with response or resistance to axi-cel (Figure 6).

Within the therapeutic-failure axis (Figure 6, top panel), lower B-cell transcript expression occurs alongside adverse transcriptional features involving *DUSP5*, *WNT11*, *CDH2*, *NCAM1*, *LRRC32* and *KLRD1*, together with exploratory Notch enrichment. These features may indicate a candidate resistance state involving altered adhesion (*CDH2*, *NCAM1*) and developmental signaling (*WNT11*), which we tentatively describe as EMT-like. This label interprets individual exploratory transcripts and is not supported by pathway-level enrichment at FDR < 0.05. *KLRD1* more likely reflects the NK/cytotoxic compartment than tumor-cell dysfunction. Of these, only *KLRD1* and *DUSP5* showed borderline FDR-supported treatment-by-gene interactions (FDR = 0.049).

Conversely, the therapeutic-sensitivity axis (Figure 6, lower panel) is characterized by higher expression of the canonical mature B-cell program, including *CD19*, *MS4A1*, *TNFRSF17*, and *IRF8,* and late-differentiation states, together with favorable associations involving *BTLA*, the CD45RA isoform (*PTPRC*), and *HLA-DOB*. Because CAR recognition is HLA-independent, *HLA-DOB* should be interpreted primarily as a marker of preserved B-cell differentiation or broader immune competence, rather than as evidence that conventional antigen presentation is required for axi-cel activity.

Overall, the model extends the emphasis from CD19 expression and baseline immune exhaustion to a broader balance between B-cell transcriptional programs and adhesion/remodeling-related transcriptional features. This framework is hypothesis-generating and highlights candidate pathways for functional and prospective validation.

## 4. Discussion

This exploratory reanalysis of the randomized ZUMA-7 trial suggests that baseline tumor transcriptional state, including but not limited to CD19 expression, is associated with outcome after axi-cel in LBCL. Although loss of *CD19* is a recognized mechanism of relapse, it accounts for only a subset of treatment failures, and progression frequently occurs in tumors that retain detectable target expression [5,6].

In the ZUMA-7 axi-cel arm, sustained responders showed higher expression of canonical B-cell lineage markers (*MS4A1*, with a borderline association for *CD19*) and of the late-differentiation marker *TNFRSF17* (BCMA), and these transcripts were associated with longer EFS. These observations recapitulate, at the gene level, the primary ZUMA-7 translational analysis, in which B-cell-related features and CD19 expression were associated with outcome after axi-cel [20], and should be read as concordance within the same dataset rather than as independent confirmation. Preservation of a mature, differentiated B-cell program may facilitate effective CAR T-cell recognition, whereas reduced lineage integrity might mark a more plastic tumor state [58,59,60]. An alternative explanation must, however, be considered. Because B-cell transcripts, and the MCP-counter B-lineage score derived from them, largely reflect the malignant B-cell content of the biopsy, lower expression in treatment failure may indicate a lower tumor-cell fraction (greater stromal or non-malignant admixture) rather than loss of lineage identity or dedifferentiation. Consistent with this possibility, most protective transcripts are expressed by B cells, whereas several transcripts higher in treatment failure (e.g., *CDH2*, *NCAM1*, *WNT11*, *C7*, *CCL21*, *PDGFA* and *NGFR*) are typically expressed by stromal or non-hematopoietic cells. Tumor cellularity was not available, so these interpretations cannot be distinguished with the present data. A crude adjustment for tumor content supported both possibilities: *CDH2* and *DUSP5* remained associated with EFS, whereas the associations of *NCAM1*, *WNT11* and *KLRD1* were attenuated (Supplementary Table S21). Notably, among the B-cell markers, only *CD19* showed a borderline treatment-by-gene interaction (FDR = 0.053), whereas *MS4A1* and *TNFRSF17* did not; B-cell lineage integrity should therefore be regarded as a candidate correlate of axi-cel outcome rather than an established, treatment-specific prerequisite. The higher MCP-counter B-lineage score in sustained responders is compatible with both interpretations.

Our findings also refine the roles of canonical immune-evasion pathways in CAR T-cell therapy. Baseline transcriptional signatures of T-cell exhaustion and global HLA downregulation did not distinguish sustained responders from patients with treatment failure in either ZUMA-7 arm. This result does not imply that immune checkpoints are biologically irrelevant. PD-1/PD-L1 and related pathways may act dynamically after infusion, directly affect the phenotype and persistence of the infused CAR T cells, or be inadequately represented by bulk pretreatment tumor RNA [7,8,61,62]. Similarly, the absence of a global association between HLA machinery and response is biologically compatible with the HLA-independent recognition conferred by the CAR construct [63]. The protective association observed for *HLA-DOB* should therefore not be interpreted as evidence that conventional antigen presentation is required for axi-cel activity; it may instead reflect preserved B-cell differentiation or a broader immunologically competent tumor state.

Our findings differ from those of Jain et al., who reported that tumor interferon signaling and suppressive myeloid cells were associated with CAR T-cell failure in large B-cell lymphoma [10]. In ZUMA-7, neither interferon-α nor interferon-γ Hallmark sets were associated with axi-cel outcome (FDR = 0.998 for both; Supplementary Table S6). Differences in cohort, line of therapy, sampling, and analytical resolution may explain this discrepancy, and the absence of a pathway-level signal in a targeted panel does not exclude a role for interferon-driven resistance programs [11]. Interestingly, two myeloid-associated transcripts (*FCN1* and *ADGRE1*) showed borderline FDR-supported interactions (FDR = 0.049), being associated with lower hazard in the SOC arm and with higher hazard in the axi-cel arm, which is compatible with a treatment-dependent role of the myeloid compartment; this observation is exploratory and requires confirmation.

Treatment failure after axi-cel was also associated, at an exploratory level, with Notch enrichment and with adhesion and tissue-remodeling transcripts. Cell-cycle programs (E2F targets and G2/M checkpoint) showed a trend toward enrichment in sustained responders after axi-cel (FDR = 0.055), whereas E2F targets and the mitotic spindle were enriched in treatment failure after SOC (FDR < 0.001 and 0.001; Supplementary Table S7). This contrast between arms was not formally tested and is hypothesis-generating: a proliferative tumor state may be compatible with effective CAR T-cell-mediated clearance, while conferring an adverse prognosis under chemotherapy. The adverse associations of *CDH2*, *NCAM1*, *LRRC32*, *DUSP5* and *WNT11* might reflect altered cell–cell or cell–matrix interactions that reduce tumor accessibility or susceptibility to CAR T-cell cytotoxicity [13,14,15]. Because lymphoma cells lack a baseline epithelial phenotype, they cannot undergo a true epithelial-to-mesenchymal transition and are better described as adopting an EMT-like plasticity [64]. In our data, this label rests on individual exploratory transcripts (*CDH2*, *NCAM1*) rather than on pathway-level enrichment (Hallmark EMT FDR = 0.194). *WNT11* has been linked to *CD79B*-mutated DLBCL and to hypoxia-related signaling [54]; however, in our data it reached only nominal significance in TCGA-DLBC (*p* = 0.036; FDR = 0.431; 9 events) and showed no evidence of treatment-by-gene interaction. *WNT11* should therefore be considered a prognostic candidate within the axi-cel arm whose specificity and generality remain unknown, rather than a pan-prognostic marker.

Conversely, *IRF8*, *BTLA*, *HLA-DOB*, *CD45RA* and canonical B-cell markers defined a transcriptional state associated with more favorable outcomes after axi-cel. *IRF8* provides a biologically plausible link between B-cell identity and immune competence, because it regulates lymphoid differentiation and interferon-responsive programs, and its disruption impairs MHC class II antigen processing in B-cell lymphoma [19,65]. The favorable association of *BTLA*, despite its classification as an inhibitory receptor, illustrates the limitations of assigning function from a single marker in bulk tissue: *BTLA* expression may identify a differentiated lymphoid compartment or a less terminally differentiated, memory-like immune state [66,67]. Because CAR recognition is HLA-independent, *HLA-DOB* is better interpreted as a marker of preserved B-cell differentiation than as evidence that conventional antigen presentation is required. More broadly, the absence of differences in MCP-counter estimates does not exclude contributions from rare populations, spatial organization, or cell–cell interactions, particularly given the incomplete marker coverage of the targeted panel.

The interaction analyses refine the interpretation of treatment specificity. Among the multivariable candidates, only *KLRD1* and *DUSP5* showed borderline FDR-supported interactions (FDR = 0.049), with nominal interactions for *CD19*, *NCAM1,* and *CD45RA*. *KLRD1* encodes CD94, which pairs with NKG2A to form an inhibitory receptor on NK and CD8^+^ T cells [47]; an NK-like transition has been described in dysfunctional CAR T cells, whereas NK-like CAR^+^ subsets were associated with remission in LBCL [48,49]. Baseline tumor *KLRD1* most likely reflects the endogenous NK/cytotoxic compartment, and its adverse association after axi-cel contrasts with a non-significant protective trend after SOC (univariable HR 0.89; *p* = 0.110; Supplementary Table S9). *DUSP5* encodes a nuclear ERK-inactivating phosphatase that modulates T-cell development and IL-2 responses [45,46]; its cellular source and functional role in LBCL are unknown. For the remaining candidates, the axi-cel-restricted pattern observed in the descriptive cross-context analyses was not supported by interaction tests, and these transcripts should not be described as predictive. Notably, most nominal interactions involved cytotoxic/NK (*KLRD1*, *CTSW*, *CD8A*, *CD244*, *PRF1*, *CCL4*, *CD247*, *XCL1/2*) or myeloid (*FCN1*, *ADGRE1*, *LILRB2*, *FCGR1A*) transcripts, all with interaction HR > 1, and most were associated with lower hazard in the SOC arm (Supplementary Table S9). This suggests that the treatment-dependent signal mainly reflects a favorable prognostic value of immune infiltration under chemotherapy that is not seen after axi-cel, in line with the primary ZUMA-7 analysis [20]. Bulk cytotoxic-lymphocyte estimates did not differ by response in the axi-cel arm (Supplementary Table S15), so this signal is unlikely to reflect overall infiltration alone.

Taken together, most associations observed in the axi-cel arm were not reproduced in the SOC arm or in TCGA-DLBC, but formal interaction testing supported a treatment-specific association for only a small subset of genes. Validation in an independent axi-cel-treated cohort with a comparator is therefore required before any of these transcripts can be considered predictive. If confirmed, tumor-state biomarkers of this kind could contribute to decisions on the sequencing of CD19-directed therapies, which currently relies largely on clinical criteria and on known mechanisms of antigen escape [22].

This study has several limitations. First, it is a retrospective secondary analysis of a single trial; concordance with the primary ZUMA-7 report reflects reuse of the same data, and no independent axi-cel-treated cohort was available after exclusion of ZUMA-1. Second, no transcript reached FDR < 0.05 in the discovery-stage differential expression analysis, and pathway signals were marginal; candidates were selected and modeled using the same outcome in the same patients, so multivariable estimates are conditional on selection and may be inflated, and several candidates (IRF8, BTLA, CDH2, NCAM1 and LRRC32) did not meet FDR < 0.05 at every stage; moreover, the survival FDR was computed within 25 candidates prefiltered on an outcome-related contrast, and none of the core genes remained FDR-supported by both tests in a panel-wide screen (Supplementary Table S20). Third, median-based dichotomization is cohort-dependent and cannot be transferred directly to clinical diagnostics; absolute, analytically validated expression cut-offs would need to be established in prospective studies. Fourth, although proportional hazards held for 12 of the 13 multivariable models, *CDH2* showed evidence of non-proportionality, and the treatment-arm and interaction terms violated this assumption in all interaction models, so interaction HRs represent averages over a median follow-up of approximately two years (23.7 and 21.6 months in the axi-cel and SOC arms) however, arm-stratified and time-split sensitivity analyses gave consistent estimates for KLRD1 and DUSP5 (Supplementary Tables S18 and S19). With 69 events in the multivariable models, model complexity was necessarily limited. Fifth, the targeted panel restricts discovery to a predefined gene set and provides incomplete coverage of MCP-counter markers and of Hallmark gene sets (e.g., 26 genes for E2F targets), so pathway results reflect only the panel-represented subset. Sixth, bulk RNA measurements cannot assign transcripts to specific cellular compartments: *KLRD1* and *NCAM1* may originate from NK or cytotoxic T cells, and *CDH2*, *WNT11,* or *LRRC32* may be expressed by stromal or regulatory immune cells rather than by the malignant clone; single-cell and spatial transcriptomic studies of pretreatment biopsies will be required to resolve this ambiguity. Seventh, TCGA-DLBC has few events and incomplete treatment annotation. Eighth, tumor cellularity was not available, so associations of B-cell transcripts may partly reflect the malignant-cell fraction of the biopsy rather than cell state; adjustment for tumor-content proxies attenuated the associations of *NCAM1*, *WNT11* and *KLRD1* (Supplementary Table S21). Ninth, the SOC arm included only 18 patients with sustained response, which limits power for response-based comparisons in that arm. Tenth, ZUMA-7 enrolled histologically heterogeneous LBCL, and subtype-specific effects could not be assessed. Finally, transcript abundance does not establish protein expression or functional activity.

Future studies should therefore combine independent validation with functional experiments. Co-culture of CD19-directed CAR T cells with DLBCL cell lines engineered to overexpress or silence candidates such as *DUSP5*, *CDH2*, *NCAM1,* or *WNT11* could test whether these genes modulate CAR T-cell activation, cytotoxicity, and persistence, and xenograft models could assess their effect on tumor control in vivo. Pharmacological modulation of the corresponding pathways (for example, MAPK/ERK or WNT signaling) and blockade of the CD94/NKG2A axis [47] could explore rational combination strategies. Overall, the results are consistent with the hypothesis that axi-cel outcome is associated with the balance between preserved B-cell transcriptional programs and adhesion/remodeling-related transcriptional features, and they provide a focused set of hypotheses for biological and clinical validation.

## 5. Conclusions

This exploratory reanalysis of the randomized ZUMA-7 trial suggests that baseline tumor transcriptional state, including but not limited to *CD19* expression, is associated with event-free survival after axi-cel in large B-cell lymphoma, whereas transcriptional markers of pre-existing T-cell exhaustion and HLA expression were not. Consistent with the primary ZUMA-7 analysis [20], preserved B-cell lineage transcripts (*MS4A1*, *TNFRSF17* and, with borderline support, *CD19*) were associated with favorable outcomes. As an incremental and hypothesis-generating contribution, gene-level candidates not individually highlighted in the primary reports (*DUSP5*, *KLRD1*, *WNT11*, *HLA-DOB* and *CD45RA* and, at a lower level of evidence, *CDH2*, *NCAM1*, *IRF8*, *BTLA* and *LRRC32*) were associated with EFS in the axi-cel arm. Formal interaction testing provided borderline support (FDR = 0.049) for a treatment-specific association only for *KLRD1* and *DUSP5*, robust to arm-stratified sensitivity models, with nominal evidence for CD19, NCAM1 and CD45RA. These findings do not establish predictive specificity; validation in independent axi-cel-treated cohorts, functional studies and prospectively defined expression thresholds are required before clinical application.

## Figures and Tables

**Table 1:** Literature context of the 13 candidate baseline transcripts associated with event-free survival after adjustment for SPD and COO in the axi-cel arm of ZUMA-7 (multivariable Cox model; Figure 4A).

Biological Domain	Marker	HR (95% CI) (Our Study)	Prior Evidence	Evidence Context
*Protective markers (HR < 1)*				
B-cell lineage/target preservation/late differentiation	*CD19*	0.82 (0.71–0.95)	Direct target of axi-cel. Low CD19 antigen density impairs CAR T-cell activity, and CD19-negative or CD19-low relapses occur after axi-cel in LBCL; B-cell lineage features were associated with favorable axi-cel outcomes in the primary ZUMA-7 analysis [6,16,20].	LBCL; anti-CD19 CAR T cells
	*MS4A1* (*CD20*)	0.84 (0.75–0.94)	Reduced CD20 expression identifies DLBCL with inferior survival after R-CHOP, and preserved B-cell lineage features were associated with axi-cel benefit in ZUMA-7 [17,20].	DLBCL (R-CHOP); LBCL (CAR T cells)
	*TNFRSF17* (*BCMA*)	0.85 (0.76–0.96)	Marks late B-cell/plasma-cell differentiation and sustains long-lived plasma-cell survival; its association parallels the B-cell features reported in ZUMA-7, but gene-level prognostic data in DLBCL are lacking [20,35].	Normal B-cell biology; LBCL (CAR T cells)
B-cell differentiation/transcriptional regulation	*IRF8*	0.78 (0.66–0.92)	In DLBCL, IRF8 transactivates CD37 and low IRF8 marks CD37-negative, poor-prognosis disease; IRF8 mutations impair MHC class II antigen processing and promote immune evasion. Conversely, high IRF8 predicted worse survival after R-CHOP, suggesting treatment-context dependence [18,19,36].	DLBCL
Antigen presentation/immune competence	*HLA-DOB*	0.78 (0.67–0.91)	HLA-DOB modulates HLA-DM-dependent peptide loading in B cells. Loss of MHC class II expression is associated with reduced tumor immunosurveillance and inferior survival in DLBCL, although HLA-DOB has not been evaluated individually [37,38,39].	DLBCL (MHC class II pathway)
Immune regulation	BTLA	0.84 (0.72–0.97)	High BTLA is associated with favorable survival in follicular lymphoma and decreases upon transformation to DLBCL, and the HVEM-BTLA axis acts as a tumor suppressor in germinal-center lymphomas. In contrast, BTLA expressed by CAR T cells restricts their efficacy, indicating compartment-dependent effects [40,41,42].	FL and transformed DLBCL; CAR T cells (preclinical)
Leukocyte isoform (B-cell and naive T-cell expressed)	*PTPRC* (CD45RA)	0.86 (0.77–0.96)	Higher naive CD45RA^+^CD62L^+^ CD4^+^ T-cell counts at apheresis were associated with complete response and longer progression-free survival after second-line CAR T-cell therapy in LBCL, and memory/naive-like CD8^+^ T cells in axi-cel products were associated with response [43,44].	LBCL; CAR T cells
*Adverse markers (HR > 1)*				
Intracellular signaling (MAPK)	*DUSP5*	1.32 (1.12–1.57)	Nuclear ERK-inactivating phosphatase with context-dependent roles across tumor types; upregulated and associated with shorter overall survival in KRAS-mutant lung adenocarcinoma. DUSP5 also modulates T-cell development and IL-2 responses. No DLBCL-specific data were identified [45,46].	Solid tumors; T cells
NK-like/cytotoxic receptor	*KLRD1* (*CD94*)	1.32 (1.11–1.58)	CD94 pairs with NKG2A to form an inhibitory checkpoint on NK and CD8^+^ T cells, and an NK-like transition marks dysfunctional CAR T cells. In LBCL, however, NK-like CAR^+^CD8^+^ subsets were associated with remission, so the cellular source of the baseline signal remains to be resolved [47,48,49].	Solid-tumor models; LBCL (CAR T cells)
Adhesion/EMT-like remodeling	*CDH2* (N-cadherin)	1.31 (1.10–1.55)	Mesenchymal cadherin linked to EMT, metastasis, and immune escape; in hematological malignancies, it mediates bone-marrow homing and adhesion-mediated drug resistance and has adverse prognostic value in multiple myeloma. No DLBCL-specific data were identified [14,50].	Pan-cancer; hematological malignancies
	*NCAM1* (*CD56*)	1.21 (1.04–1.40)	Aberrant CD56 expression occurs in a small subset (~1–7%) of DLBCL and has been associated with extranodal involvement and aggressive features; bulk NCAM1 may also reflect CD56^+^ NK cells, a compartment represented by NK-cell signatures in ZUMA-7 [20,51,52].	DLBCL (rare subset)
Developmental signaling	*WNT11*	1.35 (1.08–1.69)	Among the most up-regulated genes in CD79B-mutated DLBCL and linked to hypoxia-related signaling, but not previously associated with survival in DLBCL; tumor-intrinsic WNT/β-catenin signaling promotes T-cell exclusion in solid tumors [53,54]. In the present study, WNT11 also showed an exploratory trend in TCGA-DLBC.	DLBCL (expression); solid tumors (mechanism)
Regulatory T cell/TGF-β axis	*LRRC32* (*GARP*)	1.19 (1.00–1.42) †	GARP anchors latent TGF-β on the surface of activated FOXP3^+^ regulatory T cells. Expansion of CAR-Treg cells has been associated with non-response and relapse after axi-cel in LBCL, although LRRC32 was not assessed specifically [55,56,57].	Treg biology; LBCL (CAR T cells)

Notes. Hazard ratios (HR) and 95% confidence intervals (CI) are derived from the multivariable Cox model for event-free survival in the axi-cel arm of ZUMA-7, adjusted for baseline tumor burden (SPD) and cell of origin (COO) (Supplementary Table S10); HR < 1 indicates a protective association and HR > 1 an adverse association. The literature review prioritized DLBCL and other B-cell lymphomas, with emphasis on anti-CD19 CAR T-cell therapy; evidence from other tumor types or normal immune cells is presented only when lymphoma-specific data were unavailable, as indicated in the Evidence context column. Because the present analyses reuse the ZUMA-7 dataset, concordance with the primary ZUMA-7 report does not constitute independent validation. † Nominal significance (*p* = 0.048) with borderline false discovery rate control (FDR = 0.051). Abbreviations: BCMA: B-cell maturation antigen; CAR: chimeric antigen receptor; CI: confidence interval; DLBCL: diffuse large B-cell lymphoma; EMT: epithelial–mesenchymal transition; FL: follicular lymphoma; GARP: glycoprotein A repetitions predominant; HR: hazard ratio; LBCL: large B-cell lymphoma; MHC: major histocompatibility complex; NK: natural killer; R-CHOP: rituximab, cyclophosphamide, doxorubicin, vincristine and prednisone; TCGA: The Cancer Genome Atlas; Treg: regulatory T cell.

## Data Availability

Publicly available datasets were analyzed in this study. The ZUMA-7 data analyzed in this study are openly available in the Gene Expression Omnibus at https://www.ncbi.nlm.nih.gov/geo/ (accessed on 8 October 2026), reference number GSE248835. The TCGA-DLBC RNA-seq and clinical data presented in this study are openly available in the Genomic Data Commons Data Portal at https://portal.gdc.cancer.gov/ and in UCSC Xena (GDC hub) at https://xena.ucsc.edu/. The scripts used in this study, including those for the interaction models, proportional hazards diagnostics, reverse Kaplan–Meier follow-up, MCP-counter marker coverage, and the round-2 sensitivity analyses, are openly available in the GitHub repository at https://github.com/jeanssresende/DLBCL-CD19-Analysis (accessed on 8 October 2026).

## Supplementary Materials

The following supporting information can be downloaded at: https://www.mdpi.com/article/10.3390/medsci14060671/s1, Figure S1: Cohorts, samples and analytical workflow. Flow diagram of the ZUMA-7, TCGA-DLBC, and ZUMA-1 datasets, the exclusion of ZUMA-1, the patients and events contributing to each analysis, and the corresponding tables and figures. Figure S2: Sensitivity analyses of the gene-by-treatment interaction for the four genes with interaction FDR < 0.05 and for CD19, NCAM1, and CD45RA: (A) primary versus arm-stratified Cox model; (B) time-split interaction before and after 6 months (arm-stratified, robust standard errors). Table S1. Differential expression analysis of B-cell signature genes from the NanoString IO360 panel between the ZUMA-7 study arms. Pre-infusion (baseline) mRNA expression levels (log2) were evaluated by comparing groups with sustained response against those with treatment failure within both the axi-cel-treated cohorts and those receiving SOC. Unadjusted *p*-values derived from Wilcoxon rank-sum tests and the corresponding Benjamini–Hochberg FDR corrections are presented. Additionally, for each gene analyzed, coefficient estimates, unadjusted *p*-values, and FDR-adjusted *p*-values are provided, derived from linear regression models adjusted for COO and baseline SPD. Table S2. Analysis of gene expression associated with T-cell exhaustion and immune checkpoint signatures (PDCD1, HAVCR2, LAG3, TIGIT, CTLA4, EOMES, ENTPD1, CXCL13) across the ZUMA-7 study arms. Pre-infusion (baseline) mRNA expression levels (log2) were evaluated by comparing groups with sustained response against those with treatment failure, within both the axi-cel-treated cohorts and those receiving SOC. Unadjusted *p*-values derived from Wilcoxon rank-sum tests are presented, along with corresponding Benjamini–Hochberg FDR corrections. Additionally, for each gene analyzed, coefficient estimates, unadjusted *p*-values, and FDR-adjusted *p*-values are provided, derived from linear regression models adjusted for COO and SPD. Table S3. Gene expression analysis of genes associated with the antigen presentation machinery and class I/II human leukocyte antigen (HLA) pathways across the ZUMA-7 study arms. Pre-infusion (baseline) mRNA expression levels (log2) were evaluated by comparing groups with sustained response against those with treatment failure, within both the axi-cel-treated cohorts and those receiving SOC. Unadjusted *p*-values derived from Wilcoxon rank-sum tests are presented, along with corresponding FDR corrections using the Benjamini–Hochberg method. Additionally, for each gene feature, coefficient estimates, unadjusted *p*-values, and FDR-adjusted *p*-values are provided, derived from linear regression models adjusted for COO and SPD. Table S4. Differential gene expression analysis using the NanoString IO360 panel, comparing treatment failure and sustained response groups within the axi-cel-treated cohort of the ZUMA-7 study. Pre-infusion (baseline) mRNA expression profiles were evaluated using linear models for microarray data (limma). The table presents candidate genes from the panel that show nominal statistical significance (*p*-value < 0.05) with absolute log2 fold change values greater than |0.5| (|logFC| > 0.5). The columns represent the log2 fold change (logFC; relative expression in Failure vs. Sustained groups), the average expression across all analyzed samples (AveExpr), moderated t-statistics (t), nominal unadjusted *p*-values (*p*.Value), *p*-values adjusted via the Benjamini–Hochberg method (adj.*p*.Val/FDR), and the log-odds of differential expression (B). Table S5. Differential gene expression analysis of the NanoString IO360 panel comparing the treatment failure and sustained response groups, specifically within the standard of care (SOC) control arm of the ZUMA-7 study. Pre-infusion (baseline) mRNA expression profiles were evaluated using linear models for microarray data (limma). The table presents candidate genes from the panel that show nominal statistical significance (*p*-value < 0.05) with absolute log2 fold change values greater than |0.5| (|logFC| > 0.5). The columns represent the log2 fold change (logFC; relative expression in Failure versus Sustained groups), the average expression across all analyzed samples (AveExpr), moderated t-statistics (t), unadjusted nominal *p*-values (*p*.Value), *p*-values adjusted using the Benjamini–Hochberg method (adj.*p*.Val/FDR), and the log-odds of differential expression (B). Table S6. Gene Set Enrichment Analysis (GSEA) of MSigDB Hallmark pathways, based on differential gene expression profiles between the treatment failure and sustained response groups in the axi-cel-treated cohort of the ZUMA-7 study. Pre-infusion (baseline) mRNA expression rankings were evaluated using GSEA analytical metrics. The columns represent the biological pathway evaluated (pathway), the nominal *p*-value (pval), the Benjamini–Hochberg adjusted *p*-value (padj), the enrichment score error (log2err), the enrichment score (ES), the normalized enrichment score (NES), and the number of genes contributing to the core enrichment set (size). Table S7. Gene Set Enrichment Analysis (GSEA) of MSigDB Hallmark pathways based on differential gene expression profiles between therapeutic failure and sustained response groups specifically within the standard-of-care (SOC) control arm of the ZUMA-7 study. Pre-infusion (baseline) mRNA expression ranks were evaluated using analytical GSEA metrics. Columns represent the evaluated biological pathway (pathway), nominal *p*-value (pval), Benjamini–Hochberg adjusted *p*-value (padj), enrichment score error (log2err), enrichment score (ES), normalized enrichment score (NES), and the number of genes contributing to the core enrichment set (size). Table S8. Survival screening metrics for differentially expressed candidate genes from the NanoString IO360 panel in the axi-cel-treated cohort of the ZUMA-7 study (*p* < 0.05 and |log_2_FC| > 0.5). The columns present Kaplan–Meier log-rank test *p*-values (KM PValue), univariate Cox proportional hazards model *p*-values (UniCox PValue), univariate hazard ratios (UniCox HR), and the corresponding Benjamini–Hochberg False Discovery Rate corrections (KM FDR, UniCox FDR) for Event-Free Survival (EFS). Table S9. Survival screening metrics for differentially expressed candidate genes from the NanoString IO360 panel in the standard of care (SOC) control arm of the ZUMA-7 study (*p* < 0.05 and |log_2_FC| > 0.5). The columns present *p*-values from the Kaplan–Meier log-rank test (KM PValue), *p*-values from the univariate Cox proportional hazards model (UniCox PValue), univariate hazard ratios (UniCox HR), and the corresponding Benjamini–Hochberg False Discovery Rate (FDR) corrections (KM FDR, UniCox FDR) for Event-Free Survival (EFS). Table S10. Multivariable Cox proportional hazards analysis of prognostic candidate genes significantly associated with event-free survival (EFS) in the axi-cel-treated cohort, adjusted for baseline tumor burden (SPD) and cell-of-origin (COO) subtype (multivariable Cox *p* < 0.05; 13 of the 14 modeled genes; 121 patients, 69 events). The columns show the adjusted hazard ratio (MultiCox HR), 95% confidence intervals (lower CI, upper CI), unadjusted *p*-values (MultiCox PValue), number of patients evaluated (N patients), and Benjamini–Hochberg FDR adjustments (MultiCox FDR). Table S11. Analysis of the treatment-by-gene interaction evaluated in the combined population of the ZUMA-7 study (axi-cel versus SOC), using Cox proportional hazards models adjusted for baseline tumor burden (SPD) and cell-of-origin (COO) subtype (56 genes tested; genes with interaction *p* < 0.05 are shown). The columns present the interaction hazard ratio (interaction HR), 95% confidence intervals (lower CI, upper CI), unadjusted *p*-values (interaction *p*-value), and Benjamini–Hochberg FDR-adjusted *p*-values (interaction FDR). Table S12. Survival analyses (Kaplan–Meier with log-rank test) and Cox proportional hazards analyses of the candidate genes presented in Figure 4A (derived from the multivariable model for axi-cel; FDR values were recomputed within these 12 preselected genes and are descriptive only, the screening FDR values being those in Supplementary Table S8), evaluated in the axi-cel experimental arm, the standard-of-care (SOC) control arm of the ZUMA-7 study, and the TCGA-DLBC exploratory cohort. Table S13. Survival (Kaplan–Meier and log-rank) and Cox proportional hazards analyses of candidate genes identified from significant treatment-by-gene interactions between axi-cel and SOC (Figure 4B), evaluated in the axi-cel and SOC arms and the TCGA-DLBC exploratory cohort. Table S14. MCP-counter marker gene availability and coverage mapping within the NanoString IO360 panel. Columns display the evaluated cell population, total marker genes defined by the original MCP-counter algorithm, number of markers successfully found in the NanoString IO360 dataset (Markers Found), percentage coverage (Percent Coverage), and the specific available marker genes detected. Table S15. Evaluation of estimated immune and stromal cell populations via MCP-counter deconvolution between sustained response and failure groups within the axi-cel-treated cohort of the ZUMA-7 study. Medians of estimated abundance scores, unadjusted *p*-values, and Benjamini–Hochberg False Discovery Rate (FDR) adjustments are displayed. Table S16. Evaluation of estimated immune and stromal cell populations via MCP-counter deconvolution between sustained response and failure groups within the standard-of-care (SOC) control arm of the ZUMA-7 study. Medians of estimated abundance scores, unadjusted *p*-values, and Benjamini–Hochberg False Discovery Rate (FDR) adjustments are displayed. Table S17. Proportional hazards diagnostics (scaled Schoenfeld residuals) for the multivariable Cox models of all 14 modeled genes in the axi-cel arm, including RORC, with a note on the interaction models. Table S18. Sensitivity analyses of the gene-by-treatment interaction for all 56 candidates: primary model, arm-stratified model and arm- and COO-stratified model, with interaction HRs, *p*-values, FDR values and Schoenfeld tests. Table S19. Time-split gene-by-treatment interaction before and after 6 months for selected genes. Table S20. Panel-wide event-free survival screen in the axi-cel arm (769 mRNA features) compared with the screen within the 25 prioritized candidates. Table S21. Sensitivity analyses of the multivariable Cox models: adjustment for tumor-content proxies (MCP-counter B-lineage score and IO 360 B-cell signature) and exclusion of patients with “null” cell of origin.

## Abbreviations

The following abbreviations are used in this manuscript:

DLBCL	Diffuse large B-cell lymphoma
LBCL	Large B-cell lymphoma
R-CHOP	Rituximab, cyclophosphamide, doxorubicin, vincristine and prednisone
r/r	Relapsed or refractory
CAR	Chimeric antigen receptor
axi-cel	Axicabtagene ciloleucel
TME	Tumor microenvironment
HLA	Human leukocyte antigen
IFN	Interferon
EMT-like	Epithelial–mesenchymal transition-like
SOC	Standard of care
EFS	Event-free survival
SPD	Sum of product diameters
COO	Cell of origin
GEO	Gene Expression Omnibus
TPM	Transcripts per million
TCGA	The Cancer Genome Atlas
TCGA-DLBC	The Cancer Genome Atlas diffuse large B-cell lymphoma project
GDC	Genomic Data Commons
MCP-counter	Microenvironment Cell Populations-counter
DEA	Differential expression analysis
NES	Normalized enrichment score
MSigDB	Molecular Signatures Database
GSEA	Gene set enrichment analysis
HR	Hazard ratio
FDR	False discovery rate
OS	Overall survival
CI	Confidence interval
KM	Kaplan–Meier
BH	Benjamini–Hochberg
IO 360	NanoString PanCancer IO 360 gene expression panel
