# Supplementary material for: Baseline Tumor Transcriptional Features and Event-Free Survival After Anti-CD19 CAR T-Cell Therapy with Axicabtagene Ciloleucel in Large B-Cell Lymphoma: An Exploratory Reanalysis of the Randomized ZUMA-7 Trial
Source: Med Sci (Basel). 2026 Oct 13;14(6):671. doi: 10.3390/medsci14060671 (PMC13706688; doi:10.3390/medsci14060671)
Supplement: Supplementary file 1 [file medsci-14-00671-s001.zip › medsci-4564567-figures.zip!/Figure_S1.pdf]

Cohorts, samples and analytical workflow

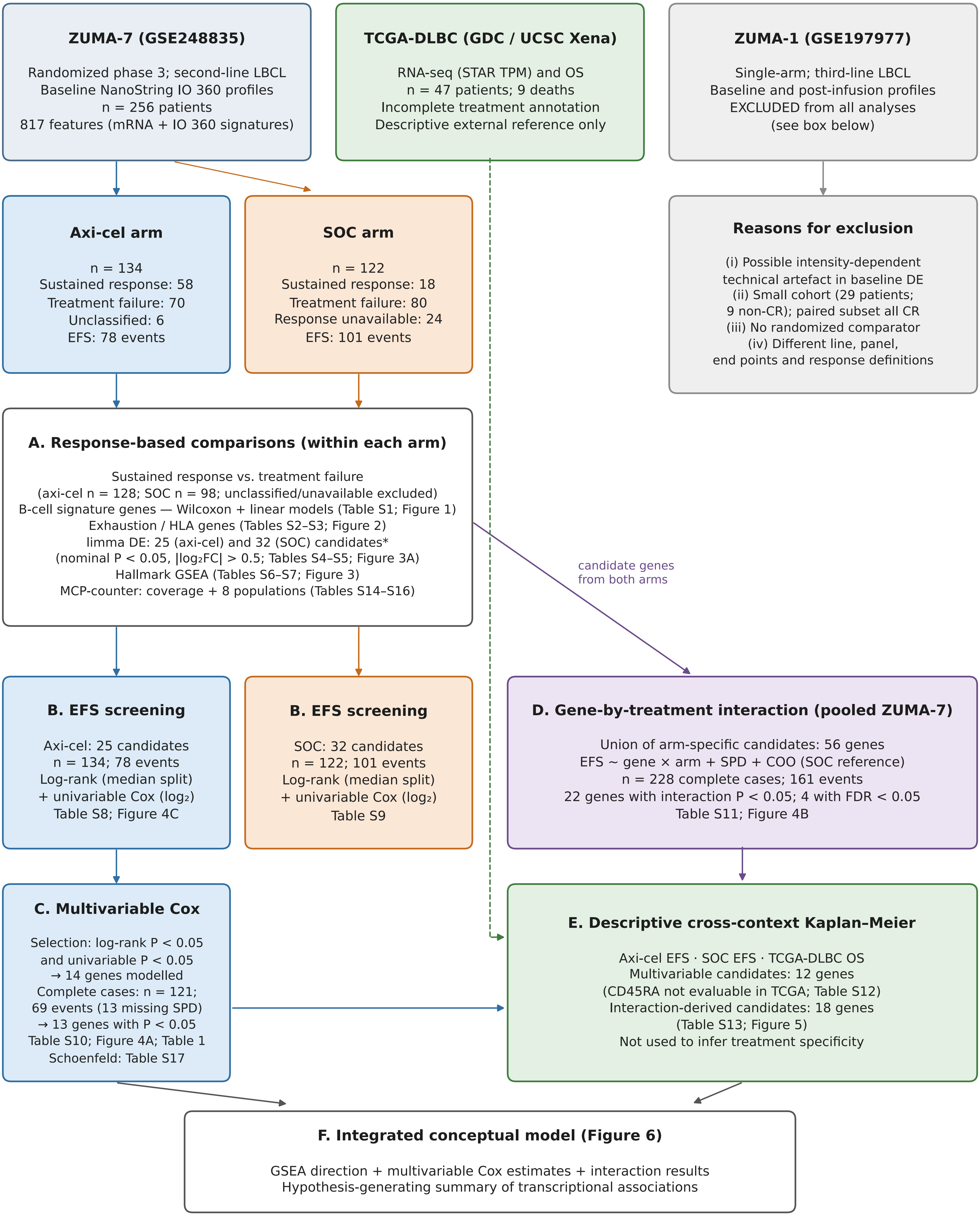

*Candidate counts follow the survival-screening input (Tables S8 and S9). LBCL, large B-cell lymphoma; SOC, standard of care; EFS, event-free survival; OS, overall survival; DE, differential expression; CR, complete response; SPD, sum of product diameters; COO, cell of origin; FDR, false discovery rate.
