# Supplementary material for: Baseline Tumor Transcriptional Features and Event-Free Survival After Anti-CD19 CAR T-Cell Therapy with Axicabtagene Ciloleucel in Large B-Cell Lymphoma: An Exploratory Reanalysis of the Randomized ZUMA-7 Trial
Source: Med Sci (Basel). 2026 Oct 13;14(6):671. doi: 10.3390/medsci14060671 (PMC13706688; doi:10.3390/medsci14060671)
Supplement: Supplementary file 1 [file medsci-14-00671-s001.zip › medsci-4564567-figures.zip!/Figure_S2.pdf]

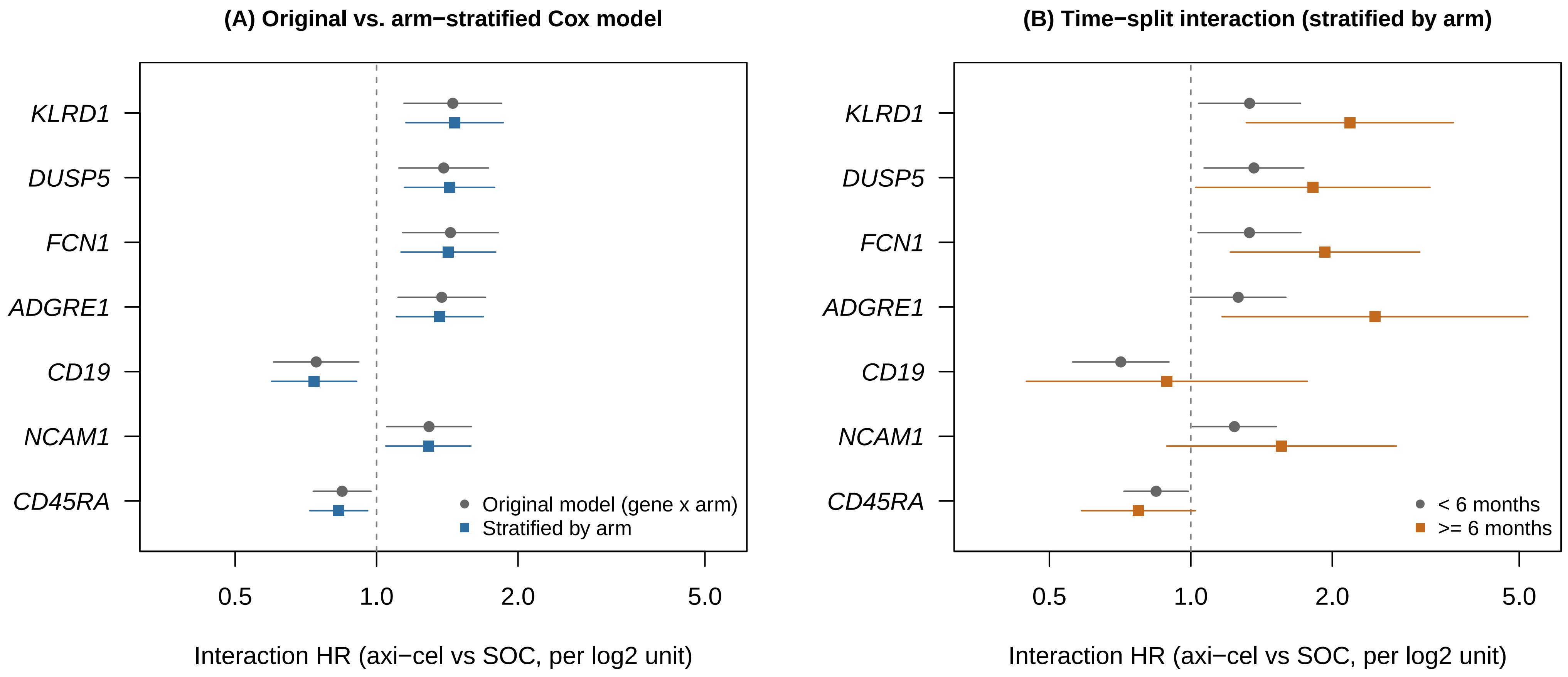
(A) Original vs. arm−stratified Cox model
KLRD1
DUSP5
FCN1
ADGRE1
CD19
NCAM1
CD45RA
Original model (gene x arm)
Stratified by arm
0.5
1.0
2.0
5.0
Interaction HR (axi−cel vs SOC, per log2 unit)
(B) Time−split interaction (stratified by arm)
KLRD1
DUSP5
FCN1
ADGRE1
CD19
NCAM1
CD45RA
< 6 months
>= 6 months
0.5
1.0
2.0
5.0
Interaction HR (axi−cel vs SOC, per log2 unit)
