# Supplementary material for: Baseline Tumor Transcriptional Features and Event-Free Survival After Anti-CD19 CAR T-Cell Therapy with Axicabtagene Ciloleucel in Large B-Cell Lymphoma: An Exploratory Reanalysis of the Randomized ZUMA-7 Trial
Source: Med Sci (Basel). 2026 Oct 13;14(6):671. doi: 10.3390/medsci14060671 (PMC13706688; doi:10.3390/medsci14060671)
Supplement: Supplementary file 1 [file medsci-14-00671-s001.zip › medsci-4564567-Tables.zip!/Supplementary_Table_S21_round2.pdf]

Supplementary Table S21. Sensitivity analyses of the multivariable Cox models for event-free survival in the axi-cel arm of ZUMA-7 (HR per log_2 unit). (A) Adjustment for tumor content: models with gene, SPD and COO (n = 121; 69 events) refitted with the MCP-counter B-lineage score or the IO 360 B-cell signature score as an additional covariate, as crude proxies for the malignant B-cell content of the biopsy. (B) Handling of cell of origin: COO was entered as a four-level category (GCB, n = 89; ABC, n = 12; unclassified, n = 15; "null" [not reported], n = 5); models were refitted after excluding the five patients with "null" COO (n = 116).

(A) Adjustment for tumor-content proxies

Gene	Base model HR (95% CI)	P	+ MCP-counter B lineage HR (95% CI)	P	+ IO 360 B-cell signature HR (95% CI)	P
CDH2	1.306 (1.103–1.548)	0.002	1.279 (1.071–1.526)	0.006	1.304 (1.090–1.561)	0.004
NCAM1	1.205 (1.038–1.398)	0.014	1.167 (0.993–1.372)	0.061	1.165 (0.995–1.364)	0.058
WNT11	1.353 (1.082–1.691)	0.008	1.224 (0.945–1.586)	0.126	1.204 (0.932–1.556)	0.155
LRRC32	1.194 (1.002–1.423)	0.048	1.213 (1.022–1.439)	0.027	1.172 (0.988–1.390)	0.068
DUSP5	1.324 (1.117–1.571)	0.001	1.275 (1.067–1.522)	0.007	1.256 (1.051–1.502)	0.012
KLRD1	1.322 (1.108–1.577)	0.002	1.240 (0.994–1.548)	0.057	1.210 (0.978–1.496)	0.079

(B) COO "null" category retained vs. excluded

Gene	HR, "null" as category (n = 121)	P	HR (95% CI), "null" excluded (n = 116)	P
DUSP5	1.324	0.001	1.402 (1.175–1.672)	<0.001
HLA-DOB	0.779	0.002	0.785 (0.671–0.919)	0.003
MS4A1	0.835	0.002	0.829 (0.738–0.932)	0.002
KLRD1	1.322	0.002	1.321 (1.098–1.590)	0.003
CDH2	1.306	0.002	1.310 (1.095–1.566)	0.003
IRF8	0.775	0.003	0.754 (0.632–0.899)	0.002
WNT11	1.353	0.008	1.352 (1.069–1.710)	0.012
CD45RA	0.858	0.008	0.822 (0.734–0.920)	<0.001
CD19	0.823	0.010	0.827 (0.707–0.967)	0.017
TNFRSF17	0.853	0.010	0.868 (0.766–0.984)	0.027
NCAM1	1.205	0.014	1.201 (1.028–1.402)	0.021
BTLA	0.837	0.020	0.852 (0.727–0.998)	0.047
LRRC32	1.194	0.048	1.207 (1.012–1.440)	0.037
RORC	1.181	0.086	1.162 (0.952–1.418)	0.139

Notes: Persistence of an association after adjustment for B-cell content is compatible with a tumor-state interpretation, whereas attenuation is compatible with confounding by tumor-cell fraction; neither proxy is a direct measure of tumor cellularity. HR, hazard ratio; CI, confidence interval; SPD, sum of product diameters; COO, cell of origin. Source: script_09_round2_sensitivity.R.
