# Supplementary material for: Baseline Tumor Transcriptional Features and Event-Free Survival After Anti-CD19 CAR T-Cell Therapy with Axicabtagene Ciloleucel in Large B-Cell Lymphoma: An Exploratory Reanalysis of the Randomized ZUMA-7 Trial
Source: Med Sci (Basel). 2026 Oct 13;14(6):671. doi: 10.3390/medsci14060671 (PMC13706688; doi:10.3390/medsci14060671)
Supplement: Supplementary file 1 [file medsci-14-00671-s001.zip › medsci-4564567-Tables.zip!/Supplementary_Table_S1_round2.pdf]

Supplementary Table S1. Differential expression analysis of B-cell signature genes from the NanoString IO360 panel between the ZUMA-7 study arms. Pre-infusion (baseline) mRNA expression levels (log_2) were evaluated by comparing groups with sustained response against those with treatment failure, within both the axi-cel-treated cohorts and those receiving SOC. Unadjusted p-values derived from Wilcoxon rank-sum tests and the corresponding Benjamini-Hochberg FDR corrections are presented. Additionally, for each gene analyzed, coefficient estimates, unadjusted p-values, and FDR-adjusted p-values are provided, derived from linear regression models adjusted for COO and baseline SPD.

Treatment	Gene	p-value (Wilcox)	Coef. estimate	P-value (Coef. Estimate)	FDR (Wilcox)	FDR (Coef. Estimate)
**axi-cel**	*BLK*	0.298	-0.41	0.128	0.383	0.192
	CD19	0.018	-0.44	0.107	0.054	0.192
	MS4A1	0.010	-1.00	0.007	0.044	0.032
	TNFRSF17	0.004	-1.07	0.004	0.036	0.032
	FCRL2	0.145	-0.82	0.057	0.249	0.128
	FAM30A	0.166	0.09	0.817	0.249	0.903
	PNOC	0.711	-0.09	0.709	0.799	0.903
	SPIB	0.831	-0.03	0.903	0.831	0.903
	TCL1A	0.102	-1.29	0.024	0.230	0.073
**SOC**	*BLK*	0.471	0.16	0.722	0.707	0.812
	CD19	0.062	1.08	0.031	0.430	0.276
	MS4A1	0.109	0.61	0.318	0.430	0.791
	TNFRSF17	0.872	-0.27	0.605	0.880	0.791
	FCRL2	0.191	0.91	0.232	0.430	0.791
	FAM30A	0.880	0.08	0.904	0.880	0.904
	PNOC	0.808	-0.18	0.615	0.880	0.791
	SPIB	0.154	0.29	0.450	0.430	0.791
	TCL1A	0.293	0.73	0.438	0.528	0.791
