# Supplementary material for: Baseline Tumor Transcriptional Features and Event-Free Survival After Anti-CD19 CAR T-Cell Therapy with Axicabtagene Ciloleucel in Large B-Cell Lymphoma: An Exploratory Reanalysis of the Randomized ZUMA-7 Trial
Source: Med Sci (Basel). 2026 Oct 13;14(6):671. doi: 10.3390/medsci14060671 (PMC13706688; doi:10.3390/medsci14060671)
Supplement: Supplementary file 1 [file medsci-14-00671-s001.zip › medsci-4564567-Tables.zip!/Supplementary_Table_S2_round2.pdf]

Supplementary Table S2. Analysis of gene expression associated with T-cell exhaustion and immune checkpoint signatures (PDCD1, HAVCR2, LAG3, TIGIT, CTLA4, EOMES, ENTPD1, CXCL13) across the ZUMA-7 study arms. Pre-infusion (baseline) mRNA expression levels (log_2) were evaluated by comparing groups with sustained response against those with treatment failure, within both the axi-cel-treated cohorts and those receiving SOC. Unadjusted p-values derived from Wilcoxon rank-sum tests are presented, along with corresponding Benjamini-Hochberg FDR corrections. Additionally, for each gene analyzed, coefficient estimates, unadjusted p-values, and FDR-adjusted p-values are provided, derived from linear regression models adjusted for COO and SPD.

Treatment	Genes	P-value (Wilcox)	FDR (Wilcox)	Coef. Estimate	P-value (Coef. Estimate)	FDR (Coef. Estimate)
axi-cel	*PDCD1*	0.272	0.987	0.451	0.100	0.541
	HAVCR2	0.941	0.987	0.099	0.615	0.702
	LAG3	0.869	0.987	0.313	0.347	0.636
	TIGIT	0.987	0.987	0.113	0.718	0.718
	CTLA4	0.567	0.987	0.284	0.407	0.636
	EOMES	0.854	0.987	0.221	0.477	0.636
	ENTPD1	0.594	0.987	0.328	0.135	0.541
	CXCL13	0.651	0.987	0.495	0.282	0.636
SOC	*PDCD1*	0.306	0.613	-0.440	0.333	0.829
	HAVCR2	0.156	0.613	-0.214	0.481	0.829
	LAG3	0.238	0.613	-0.669	0.240	0.829
	TIGIT	0.656	0.724	-0.236	0.602	0.829
	CTLA4	0.676	0.724	-0.106	0.829	0.829
	EOMES	0.185	0.613	-0.569	0.253	0.829
	ENTPD1	0.724	0.724	0.079	0.823	0.829
	CXCL13	0.500	0.724	-0.206	0.772	0.829
