# Supplementary material for: Baseline Tumor Transcriptional Features and Event-Free Survival After Anti-CD19 CAR T-Cell Therapy with Axicabtagene Ciloleucel in Large B-Cell Lymphoma: An Exploratory Reanalysis of the Randomized ZUMA-7 Trial
Source: Med Sci (Basel). 2026 Oct 13;14(6):671. doi: 10.3390/medsci14060671 (PMC13706688; doi:10.3390/medsci14060671)
Supplement: Supplementary file 1 [file medsci-14-00671-s001.zip › medsci-4564567-Tables.zip!/Supplementary_Table_S3_round2.pdf]

Supplementary Table S3. Gene expression analysis of genes associated with the antigen presentation machinery and class I/II human leukocyte antigen (HLA) pathways across the ZUMA-7 study arms. Pre-infusion (baseline) mRNA expression levels (log_2) were evaluated by comparing groups with sustained response against those with treatment failure, within both the axi-cel-treated cohorts and those receiving SOC. Unadjusted p-values derived from Wilcoxon rank-sum tests are presented, along with corresponding FDR corrections using the Benjamini-Hochberg method. Additionally, for each gene feature, coefficient estimates, unadjusted p-values, and FDR-adjusted p-values are provided, derived from linear regression models adjusted for COO and SPD.

Treatment	Genes	P-value (Wilcox)	Coef. Estimate	P-value (Coef. Estimate)	FDR (Wilcox)	FDR (Coef. Estimate)
axi-cel	*HLA-A*	0.156	-0.254	0.562	0.854	0.944
	HLA-B	0.454	0.030	0.866	0.854	0.962
	HLA-C	0.964	0.110	0.605	0.964	0.944
	B2M	0.641	0.132	0.419	0.854	0.944
	TAP1	0.307	-0.130	0.488	0.854	0.944
	TAP2	0.347	-0.160	0.260	0.854	0.944
	TAPBP	0.587	-0.087	0.465	0.854	0.944
	PSMB8	0.914	-0.130	0.362	0.964	0.944
	PSMB9	0.529	-0.168	0.359	0.854	0.944
	PSMB10	0.604	0.056	0.614	0.854	0.944
	NLRC5	0.564	0.200	0.302	0.854	0.944
	HLA-DRA	0.802	0.059	0.799	0.964	0.962
	HLA-DRB1	0.477	0.241	0.313	0.854	0.944
	HLA-DRB5	0.302	0.728	0.374	0.854	0.944
	HLA-DPA1	0.641	0.075	0.721	0.854	0.962
	HLA-DPB1	0.529	0.001	0.998	0.854	0.998
	HLA-DQA1	0.926	-0.177	0.847	0.964	0.962
	HLA-DQB1	0.561	-0.061	0.930	0.854	0.979
	HLA-E	0.614	0.045	0.778	0.854	0.962
	HLA-F	0.960	0.145	0.488	0.964	0.944
SOC	*HLA-A*	0.154	-0.404	0.468	0.615	0.996
	HLA-B	0.120	-0.236	0.401	0.615	0.996
	HLA-C	0.333	-0.010	0.974	0.652	0.996
	B2M	0.154	-0.319	0.230	0.615	0.996
	TAP1	0.124	-0.191	0.502	0.615	0.996
	TAP2	0.127	-0.092	0.631	0.615	0.996
	TAPBP	0.391	-0.047	0.799	0.652	0.996
	PSMB8	0.703	0.083	0.713	0.827	0.996
	PSMB9	0.604	-0.014	0.958	0.827	0.996
	PSMB10	0.670	0.037	0.855	0.827	0.996
	NLRC5	0.500	-0.001	0.996	0.769	0.996
	HLA-DRA	0.391	-0.191	0.578	0.652	0.996

HLA-DRB1	0.265	-0.340	0.429	0.652	0.996
HLA-DRB5	0.228	2.689	0.042	0.650	0.847
HLA-DPA1	0.909	-0.032	0.930	0.909	0.996
HLA-DPB1	0.710	-0.080	0.831	0.827	0.996
HLA-DQA1	0.745	1.304	0.372	0.827	0.996
HLA-DQB1	0.808	0.716	0.515	0.850	0.996
HLA-E	0.347	-0.064	0.814	0.652	0.996
HLA-F	0.217	-0.120	0.707	0.650	0.996
