# Supplementary material for: Baseline Tumor Transcriptional Features and Event-Free Survival After Anti-CD19 CAR T-Cell Therapy with Axicabtagene Ciloleucel in Large B-Cell Lymphoma: An Exploratory Reanalysis of the Randomized ZUMA-7 Trial
Source: Med Sci (Basel). 2026 Oct 13;14(6):671. doi: 10.3390/medsci14060671 (PMC13706688; doi:10.3390/medsci14060671)
Supplement: Supplementary file 1 [file medsci-14-00671-s001.zip › medsci-4564567-Tables.zip!/Supplementary_Table_S4_round2.pdf]

**Supplementary Table S4.** Differential gene expression analysis using the NanoString IO360 panel, comparing treatment failure and sustained response groups within the axi-cel-treated cohort of the ZUMA-7 study. Pre-infusion (baseline) mRNA expression profiles were evaluated using linear models for microarray data (limma). The table presents candidate genes from the panel that show nominal statistical significance (P.Value < 0.05) with absolute log2 fold change values greater than |0.5| (|logFC| > 0.5). The columns represent the log2 fold change (logFC; relative expression in Failure vs. Sustained groups), the average expression across all analyzed samples (AveExpr), moderated t-statistics (t), nominal unadjusted p-values (P.Value), p-values adjusted via the Benjamini-Hochberg method (adj.P.Val / FDR), and the log-odds of differential expression (B).

| Gene | logFC | AveExpr | t | P.Value | adj.P.Val | B |
|---|---|---|---|---|---|---|
| *MET* | 0.86 | 6.25 | 3.19 | 0.002 | 0.600 | -3.43 |
| *KLRD1* | 0.69 | 5.50 | 3.01 | 0.003 | 0.600 | -3.56 |
| *TNFRSF17* | -0.96 | 7.41 | -2.92 | 0.004 | 0.600 | -3.63 |
| *TNFRSF25* | 0.56 | 6.38 | 2.88 | 0.005 | 0.600 | -3.66 |
| *PDGFA* | 0.51 | 5.59 | 2.85 | 0.005 | 0.600 | -3.68 |
| *DUSP5* | 0.68 | 7.76 | 2.83 | 0.005 | 0.600 | -3.70 |
| *RORC* | 0.54 | 3.15 | 2.75 | 0.007 | 0.600 | -3.75 |
| *WNT11* | 0.52 | 3.79 | 2.75 | 0.007 | 0.600 | -3.75 |
| *CDH2* | 0.61 | 5.63 | 2.68 | 0.008 | 0.600 | -3.80 |
| *NCAM1* | 0.66 | 4.10 | 2.67 | 0.009 | 0.600 | -3.81 |
| *EPCAM* | 0.72 | 4.85 | 2.64 | 0.009 | 0.600 | -3.83 |
| *C7* | 0.99 | 6.64 | 2.64 | 0.009 | 0.600 | -3.83 |
| *MS4A1* | -0.82 | 11.72 | -2.52 | 0.013 | 0.600 | -3.91 |
| *MAML3* | 0.65 | 7.28 | 2.46 | 0.015 | 0.600 | -3.94 |
| *IRF8* | -0.52 | 10.56 | -2.46 | 0.015 | 0.600 | -3.95 |
| *NGFR* | 0.53 | 5.41 | 2.43 | 0.017 | 0.600 | -3.97 |
| *CCL21* | 1.12 | 7.74 | 2.39 | 0.018 | 0.605 | -3.99 |
| *LRRC32* | 0.53 | 6.99 | 2.30 | 0.023 | 0.626 | -4.04 |
| *BTLA* | -0.64 | 5.86 | -2.25 | 0.026 | 0.626 | -4.08 |
| *CPA3* | 0.56 | 4.70 | 2.24 | 0.027 | 0.626 | -4.08 |
| *HLA-DOB* | -0.61 | 8.56 | -2.21 | 0.029 | 0.626 | -4.10 |
| *KLRB1* | 0.55 | 5.89 | 2.13 | 0.035 | 0.626 | -4.14 |
| *CD45RA* | -0.71 | 9.77 | -2.09 | 0.038 | 0.626 | -4.16 |
| *CD19* | -0.51 | 10.47 | -2.08 | 0.040 | 0.626 | -4.17 |
| *SERPINA9* | 1.10 | 7.25 | 2.01 | 0.047 | 0.626 | -4.21 |
