# Supplementary material for: Baseline Tumor Transcriptional Features and Event-Free Survival After Anti-CD19 CAR T-Cell Therapy with Axicabtagene Ciloleucel in Large B-Cell Lymphoma: An Exploratory Reanalysis of the Randomized ZUMA-7 Trial
Source: Med Sci (Basel). 2026 Oct 13;14(6):671. doi: 10.3390/medsci14060671 (PMC13706688; doi:10.3390/medsci14060671)
Supplement: Supplementary file 1 [file medsci-14-00671-s001.zip › medsci-4564567-Tables.zip!/Supplementary_Table_S5_round2.pdf]

Supplementary Table S5. Differential gene expression analysis of the NanoString IO360 panel comparing the treatment failure and sustained response groups, specifically within the standard of care (SOC) control arm of the ZUMA-7 study. Pre-infusion (baseline) mRNA expression profiles were evaluated using linear models for microarray data (limma). The table presents candidate genes from the panel that show nominal statistical significance (P.Value < 0.05) with absolute $\log_2$ fold change values greater than |0.5| (|logFC| > 0.5). The columns represent the $\log_2$ fold change (logFC; relative expression in Failure versus Sustained groups), the average expression across all analyzed samples (AveExpr), moderated t-statistics (t), unadjusted nominal p-values (P.Value), p-values adjusted using the Benjamini-Hochberg method (adj.P.Val / FDR), and the log-odds of differential expression (B).

Gene	logFC	AveExpr	t	P.Value	adj.P.Val	B
PRF1	-1.05	7.09	-2.78	0.006	0.800	-4.15
MAGEA4	-1.20	4.12	-2.57	0.012	0.800	-4.22
KIR3DL2	-0.89	3.67	-2.56	0.012	0.800	-4.23
CTAG1B	-0.94	2.70	-2.55	0.012	0.800	-4.23
CD68	-0.56	10.06	-2.49	0.014	0.800	-4.25
CD14	-0.80	9.21	-2.47	0.015	0.800	-4.26
CEBPB	-0.60	9.45	-2.43	0.017	0.800	-4.27
CD8A	-0.91	7.78	-2.42	0.017	0.800	-4.27
C2	-0.75	7.49	-2.39	0.019	0.800	-4.28
KLRD1	-0.77	5.84	-2.35	0.021	0.800	-4.29
MAGEA1	-1.02	4.97	-2.33	0.022	0.800	-4.30
NFAM1	-0.63	6.31	-2.32	0.022	0.800	-4.30
CCL4	-0.65	8.22	-2.30	0.023	0.800	-4.31
CD244	-0.67	4.79	-2.28	0.025	0.800	-4.31
FCGR1A	-0.83	7.10	-2.26	0.026	0.800	-4.32
SLC7A5	-0.54	8.42	-2.26	0.026	0.800	-4.32
FCN1	-0.69	7.24	-2.23	0.028	0.800	-4.33
CXCL16	-0.58	7.14	-2.21	0.029	0.800	-4.33
ADGRE1	-0.74	4.59	-2.20	0.030	0.800	-4.34
CCND1	-0.66	7.37	-2.18	0.031	0.800	-4.34
SHC2	-0.70	4.74	-2.17	0.032	0.800	-4.34
LILRB2	-0.63	7.31	-2.16	0.033	0.800	-4.35
ITGB2	-0.60	11.28	-2.16	0.033	0.800	-4.35
IL32	-0.77	10.31	-2.15	0.034	0.800	-4.35
HSD11B1	-0.85	7.02	-2.15	0.034	0.800	-4.35
CTSW	-0.66	5.95	-2.10	0.038	0.800	-4.37
CMKLR1	-0.50	8.75	-2.07	0.041	0.800	-4.37
XCL1/2	-0.66	5.49	-2.07	0.041	0.800	-4.37
HLA-DRB5	2.28	5.76	2.07	0.041	0.800	-4.38
CD247	-0.70	6.09	-2.05	0.043	0.800	-4.38
NKG7	-0.90	9.83	-2.03	0.045	0.800	-4.38
CSF1	-0.60	7.17	-2.00	0.049	0.800	-4.39
