# Supplementary material for: Baseline Tumor Transcriptional Features and Event-Free Survival After Anti-CD19 CAR T-Cell Therapy with Axicabtagene Ciloleucel in Large B-Cell Lymphoma: An Exploratory Reanalysis of the Randomized ZUMA-7 Trial
Source: Med Sci (Basel). 2026 Oct 13;14(6):671. doi: 10.3390/medsci14060671 (PMC13706688; doi:10.3390/medsci14060671)
Supplement: Supplementary file 1 [file medsci-14-00671-s001.zip › medsci-4564567-Tables.zip!/Supplementary_Table_S6_round2.pdf]

Supplementary Table S6. Gene Set Enrichment Analysis (GSEA) of MSigDB Hallmark pathways, based on differential gene expression profiles between the treatment failure and sustained response groups in the axi-cel-treated cohort of the ZUMA-7 study. Pre-infusion (baseline) mRNA expression rankings were evaluated using GSEA analytical metrics. The columns represent the biological pathway evaluated (pathway), the nominal p-value (pval), the Benjamini-Hochberg adjusted p-value (padj), the enrichment score error (log_2err), the enrichment score (ES), the normalized enrichment score (NES), and the number of genes contributing to the core enrichment set (size).

pathway	pval	padj	log2err	ES	NES	size
HALLMARK NOTCH SIGNALING	0.002	0.055	0.43	0.71	1.74	11
HALLMARK E2F TARGETS	0.004	0.055	0.41	-0.43	-1.81	26
HALLMARK G2M CHECKPOINT	0.005	0.055	0.41	-0.51	-1.94	18
HALLMARK MYOGENESIS	0.014	0.114	0.38	0.62	1.56	12
HALLMARK WNT BETA CATENIN SIGNALING	0.035	0.194	0.27	0.55	1.49	15
HALLMARK KRAS SIGNALING DN	0.040	0.194	0.25	0.54	1.48	16
HALLMARK EPITHELIAL MESENCHYMAL TRANSITION	0.041	0.194	0.23	0.42	1.43	52
HALLMARK ANGIOGENESIS	0.054	0.221	0.21	0.55	1.46	14
HALLMARK UV RESPONSE DN	0.121	0.391	0.14	0.48	1.34	19
HALLMARK P53 PATHWAY	0.133	0.391	0.12	0.42	1.30	29
HALLMARK GLYCOLYSIS	0.133	0.391	0.13	0.43	1.30	26
HALLMARK MITOTIC SPINDLE	0.142	0.391	0.27	-0.41	-1.31	11
HALLMARK PI3K AKT MTOR SIGNALING	0.260	0.660	0.09	0.41	1.17	22
HALLMARK TNFA SIGNALING VIA NFKB	0.339	0.799	0.07	0.31	1.08	72
HALLMARK ALLOGRAFT REJECTION	0.390	0.858	0.20	-0.19	-1.11	110
HALLMARK ESTROGEN RESPONSE LATE	0.506	0.884	0.05	0.34	0.99	22
HALLMARK HYPOXIA	0.524	0.884	0.05	0.30	0.97	37
HALLMARK KRAS SIGNALING UP	0.526	0.884	0.05	0.30	0.97	38
HALLMARK UV RESPONSE UP	0.535	0.884	0.05	0.34	0.97	20
HALLMARK COAGULATION	0.541	0.884	0.05	0.34	0.97	19
HALLMARK APICAL JUNCTION	0.568	0.884	0.04	0.31	0.94	31
HALLMARK ESTROGEN RESPONSE EARLY	0.590	0.884	0.05	0.34	0.93	17
HALLMARK INFLAMMATORY RESPONSE	0.686	0.984	0.03	0.25	0.90	80

HALLMARK XENOBIOTIC METABOLISM	0.777	0.998	0.03	0.28	0.77	17
HALLMARK TGF BETA SIGNALING	0.826	0.998	0.10	-0.25	-0.76	10
HALLMARK ADIPOGENESIS	0.882	0.998	0.03	0.27	0.67	11
HALLMARK INTERFERON ALPHA RESPONSE	0.916	0.998	0.02	0.21	0.69	42
HALLMARK COMPLEMENT	0.916	0.998	0.02	0.21	0.66	36
HALLMARK IL6 JAK STAT3 SIGNALING	0.922	0.998	0.02	0.21	0.69	47
HALLMARK IL2_ STAT5 SIGNALING	0.926	0.998	0.02	0.20	0.69	56
HALLMARK MTORC1 SIGNALING	0.967	0.998	0.11	-0.16	-0.62	22
HALLMARK APOPTOSIS	0.978	0.998	0.01	0.17	0.56	43
HALLMARK INTERFERON GAMMA RESPONSE	0.998	0.998	0.01	0.14	0.50	86
