# Supplementary material for: Baseline Tumor Transcriptional Features and Event-Free Survival After Anti-CD19 CAR T-Cell Therapy with Axicabtagene Ciloleucel in Large B-Cell Lymphoma: An Exploratory Reanalysis of the Randomized ZUMA-7 Trial
Source: Med Sci (Basel). 2026 Oct 13;14(6):671. doi: 10.3390/medsci14060671 (PMC13706688; doi:10.3390/medsci14060671)
Supplement: Supplementary file 1 [file medsci-14-00671-s001.zip › medsci-4564567-Tables.zip!/Supplementary_Table_S7_round2.pdf]

Supplementary Table S7. Gene Set Enrichment Analysis (GSEA) of MSigDB Hallmark pathways based on differential gene expression profiles between therapeutic failure and sustained response groups specifically within the standard-of-care (SOC) control arm of the ZUMA-7 study. Pre-infusion (baseline) mRNA expression ranks were evaluated using analytical GSEA metrics. Columns represent the evaluated biological pathway (pathway), nominal p-value (pval), Benjamini-Hochberg adjusted p-value (padj), enrichment score error (log_2err), enrichment score (ES), normalized enrichment score (NES), and the number of genes contributing to the core enrichment set (size).

pathway	pval	padj	log2err	ES	NES	size
HALLMARK E2F TARGETS	2.33E-09	7.69E-08	0.775	0.741	3.159	26
HALLMARK MITOTIC SPINDLE	3.67E-05	0.001	0.557	0.783	2.552	11
HALLMARK G2M CHECKPOINT	0.010	0.072	0.381	0.442	1.653	18
HALLMARK ALLOGRAFT REJECTION	0.010	0.072	0.381	-0.385	-1.428	110
HALLMARK XENOBIOTIC METABOLISM	0.011	0.072	0.381	-0.576	-1.606	17
HALLMARK INFLAMMATORY RESPONSE	0.014	0.078	0.381	-0.406	-1.466	80
HALLMARK MYOGENESIS	0.031	0.148	0.288	-0.592	-1.522	12
HALLMARK IL6 JAK STAT3 SIGNALING	0.053	0.217	0.200	-0.408	-1.383	47
HALLMARK ADIPOGENESIS	0.082	0.278	0.175	-0.548	-1.381	11
HALLMARK TNFA SIGNALING VIA NFKB	0.084	0.278	0.153	-0.361	-1.289	72
HALLMARK COMPLEMENT	0.096	0.289	0.148	-0.410	-1.329	36
HALLMARK INTERFERON GAMMA RESPONSE	0.109	0.300	0.132	-0.343	-1.246	86
HALLMARK TGF BETA SIGNALING	0.129	0.329	0.249	0.429	1.339	10
HALLMARK NOTCH SIGNALING	0.170	0.401	0.117	-0.496	-1.249	11
HALLMARK ESTROGEN RESPONSE LATE	0.213	0.447	0.097	-0.413	-1.221	22
HALLMARK COAGULATION	0.217	0.447	0.096	-0.423	-1.217	19
HALLMARK EPITHELIAL MESENCHYMAL TRANSITION	0.247	0.479	0.082	-0.336	-1.148	52

HALLMARK ANGIOGENESIS	0.320	0.587	0.077	-0.425	-1.136	14
HALLMARK APICAL JUNCTION	0.385	0.669	0.064	-0.338	-1.068	31
HALLMARK KRAS SIGNALING UP	0.409	0.674	0.059	-0.322	-1.053	38
HALLMARK PI3K AKT MTOR SIGNALING	0.503	0.790	0.052	-0.334	-0.988	22
HALLMARK MTORC1 SIGNALING	0.577	0.834	0.046	-0.317	-0.939	22
HALLMARK UV RESPONSE UP	0.588	0.834	0.045	-0.319	-0.930	20
HALLMARK ESTROGEN RESPONSE EARLY	0.624	0.834	0.044	-0.326	-0.910	17
HALLMARK P53 PATHWAY	0.632	0.834	0.041	-0.290	-0.908	29
HALLMARK UV RESPONSE DN	0.683	0.867	0.039	-0.299	-0.862	19
HALLMARK GLYCOLYSIS	0.730	0.893	0.034	-0.273	-0.834	26
HALLMARK KRAS SIGNALING DN	0.808	0.908	0.032	-0.269	-0.741	16
HALLMARK HYPOXIA	0.821	0.908	0.027	-0.238	-0.776	37
HALLMARK APOPTOSIS	0.825	0.908	0.025	-0.230	-0.771	43
HALLMARK INTERFERON ALPHA RESPONSE	0.889	0.946	0.175	0.166	0.788	42
HALLMARK IL2 STAT5 SIGNALING	0.954	0.955	0.014	-0.187	-0.646	56
HALLMARK WNT BETA CATENIN SIGNALING	0.955	0.955	0.024	-0.214	-0.583	15
