# Supplementary material for: Baseline Tumor Transcriptional Features and Event-Free Survival After Anti-CD19 CAR T-Cell Therapy with Axicabtagene Ciloleucel in Large B-Cell Lymphoma: An Exploratory Reanalysis of the Randomized ZUMA-7 Trial
Source: Med Sci (Basel). 2026 Oct 13;14(6):671. doi: 10.3390/medsci14060671 (PMC13706688; doi:10.3390/medsci14060671)
Supplement: Supplementary file 1 [file medsci-14-00671-s001.zip › medsci-4564567-Tables.zip!/Supplementary_Table_S8_round2.pdf]

Supplementary Table S8. Survival screening metrics for differentially expressed candidate genes from the NanoString IO360 panel in the axi-cel-treated cohort of the ZUMA-7 study (P < 0.05 and $|log_2FC| > 0.5$). The columns present Kaplan-Meier log-rank test p-values (KM PValue), univariate Cox proportional hazards model p-values (UniCox PValue), univariate hazard ratios (UniCox HR), and the corresponding Benjamini-Hochberg False Discovery Rate corrections (KM FDR, UniCox FDR) for Event-Free Survival (EFS).

Gene	KM PValue	UniCox PValue	UniCox HR	KM FDR	UniCox FDR
MET	0.064	0.006	1.19	0.093	0.015
KLRD1	0.006	0.002	1.29	0.024	0.014
TNFRSF17	0.010	0.012	0.86	0.031	0.023
TNFRSF25	0.151	0.012	1.30	0.189	0.023
PDGFA	0.372	0.017	1.30	0.387	0.031
DUSP5	0.001	2.26E-04	1.34	0.006	0.006
RORC	0.020	0.024	1.23	0.045	0.040
WNT11	0.005	0.003	1.35	0.024	0.014
CDH2	0.043	0.035	1.18	0.068	0.054
NCAM1	0.037	0.045	1.16	0.062	0.059
EPCAM	0.599	0.004	1.23	0.599	0.014
C7	0.136	0.040	1.11	0.179	0.055
MS4A1	3.75E-05	0.002	0.85	0.001	0.014
MAML3	0.219	0.164	1.11	0.249	0.164
IRF8	0.035	0.005	0.80	0.062	0.015
NGFR	0.031	0.061	1.18	0.062	0.073
CCL21	0.019	0.075	1.07	0.045	0.085
LRRC32	0.019	0.038	1.18	0.045	0.055
BTLA	0.034	0.007	0.82	0.062	0.016
CPA3	0.343	0.054	1.16	0.373	0.068
HLA-DOB	0.009	0.001	0.80	0.031	0.011
KLRB1	0.136	0.104	1.13	0.179	0.113
CD45RA	0.001	0.006	0.87	0.006	0.015
CD19	0.005	0.004	0.81	0.024	0.014
SERPINA9	0.196	0.135	1.06	0.234	0.141
