# Supplementary material for: Baseline Tumor Transcriptional Features and Event-Free Survival After Anti-CD19 CAR T-Cell Therapy with Axicabtagene Ciloleucel in Large B-Cell Lymphoma: An Exploratory Reanalysis of the Randomized ZUMA-7 Trial
Source: Med Sci (Basel). 2026 Oct 13;14(6):671. doi: 10.3390/medsci14060671 (PMC13706688; doi:10.3390/medsci14060671)
Supplement: Supplementary file 1 [file medsci-14-00671-s001.zip › medsci-4564567-Tables.zip!/Supplementary_Table_S9_round2.pdf]

Supplementary Table S9. Survival screening metrics for differentially expressed candidate genes from the NanoString IO360 panel in the standard of care (SOC) control arm of the ZUMA-7 study ($P < 0.05$ and |log2FC| > 0.5). The columns present p-values from the Kaplan-Meier log-rank test (KM PValue), p-values from the univariate Cox proportional hazards model (UniCox PValue), univariate hazard ratios (UniCox HR), and the corresponding Benjamini-Hochberg False Discovery Rate (FDR) corrections (KM FDR, UniCox FDR) for Event-Free Survival (EFS).

Gene	KM PValue	UniCox PValue	UniCox HR	KM FDR	UniCox FDR
PRF1	0.124	0.011	0.84	0.241	0.072
MAGEA4	0.407	0.036	0.88	0.482	0.085
KIR3DL2	0.055	0.064	0.87	0.211	0.085
CTAG1B	0.731	0.063	0.87	0.780	0.085
CD68	0.245	0.035	0.80	0.341	0.085
CD14	0.060	0.009	0.80	0.211	0.072
CEBPB	0.018	0.094	0.84	0.094	0.111
CD8A	0.076	0.056	0.88	0.211	0.085
C2	0.308	0.062	0.87	0.394	0.085
KLRD1	0.337	0.110	0.89	0.415	0.121
MAGEA1	0.306	0.194	0.92	0.394	0.207
NFAM1	0.013	0.011	0.80	0.094	0.072
CCL4	0.108	0.053	0.84	0.241	0.085
CD244	0.648	0.052	0.85	0.715	0.085
FCGR1A	0.074	0.040	0.86	0.211	0.085
SLC7A5	0.531	0.351	0.90	0.607	0.351
FCN1	0.054	0.010	0.80	0.211	0.072
CXCL16	0.114	0.053	0.82	0.241	0.085
ADGRE1	0.011	0.045	0.85	0.094	0.085
CCND1	0.128	0.069	0.83	0.241	0.085
SHC2	0.002	0.022	0.82	0.035	0.085
LILRB2	0.098	0.028	0.81	0.241	0.085
ITGB2	0.001	0.006	0.79	0.035	0.072
IL32	0.142	0.029	0.86	0.242	0.085
HSD11B1	0.079	0.017	0.85	0.211	0.085
CTSW	0.186	0.019	0.82	0.291	0.085
CMKLR1	0.016	0.040	0.81	0.094	0.085
XCL1/2	0.781	0.100	0.86	0.806	0.114
HLA-DRB5	0.858	0.250	1.03	0.858	0.258
CD247	0.221	0.032	0.85	0.322	0.085
NKG7	0.144	0.044	0.89	0.242	0.085
CSF1	0.191	0.066	0.85	0.291	0.085
