# Supplementary material for: Baseline Tumor Transcriptional Features and Event-Free Survival After Anti-CD19 CAR T-Cell Therapy with Axicabtagene Ciloleucel in Large B-Cell Lymphoma: An Exploratory Reanalysis of the Randomized ZUMA-7 Trial
Source: Med Sci (Basel). 2026 Oct 13;14(6):671. doi: 10.3390/medsci14060671 (PMC13706688; doi:10.3390/medsci14060671)
Supplement: Supplementary file 1 [file medsci-14-00671-s001.zip › medsci-4564567-Tables.zip!/Supplementary_Table_S10_round2.pdf]

Supplementary Table S10. Multivariate Cox proportional hazards analysis of prognostic candidate genes significantly associated with event-free survival (EFS) in the axi-cel-treated cohort, adjusted for baseline tumor burden (SPD) and cell-of-origin (COO) subtype (multivariate Cox p-value < 0.05). The columns show the adjusted hazard ratio (MultiCox HR), 95% confidence intervals (lower CI, upper CI), unadjusted p-values (MultiCox PValue), number of patients evaluated (N patients), and Benjamini-Hochberg FDR adjustments (MultiCox FDR).

Gene	MultiCox HR	CI lower	CI upper	MultiCox PValue	N patients	MultiCox FDR
DUSP5	1.324	1.12	1.57	0.001	121	0.006
HLA-DOB	0.779	0.67	0.91	0.002	121	0.006
MS4A1	0.835	0.75	0.94	0.002	121	0.006
KLRD1	1.322	1.11	1.58	0.002	121	0.006
CDH2	1.306	1.10	1.55	0.002	121	0.006
IRF8	0.775	0.66	0.92	0.003	121	0.006
WNT11	1.353	1.08	1.69	0.008	121	0.014
CD45RA	0.858	0.77	0.96	0.008	121	0.014
CD19	0.823	0.71	0.95	0.010	121	0.014
TNFRSF17	0.853	0.76	0.96	0.010	121	0.014
NCAM1	1.205	1.04	1.40	0.014	121	0.018
BTLA	0.837	0.72	0.97	0.020	121	0.023
LRRC32	1.194	1.00	1.42	0.048	121	0.051
