# Supplementary material for: Baseline Tumor Transcriptional Features and Event-Free Survival After Anti-CD19 CAR T-Cell Therapy with Axicabtagene Ciloleucel in Large B-Cell Lymphoma: An Exploratory Reanalysis of the Randomized ZUMA-7 Trial
Source: Med Sci (Basel). 2026 Oct 13;14(6):671. doi: 10.3390/medsci14060671 (PMC13706688; doi:10.3390/medsci14060671)
Supplement: Supplementary file 1 [file medsci-14-00671-s001.zip › medsci-4564567-Tables.zip!/Supplementary_Table_S11_round2.pdf]

Supplementary Table S11. Analysis of the treatment-by-gene interaction evaluated in the combined population of the ZUMA-7 study (axi-cel versus SOC), using Cox proportional hazards models adjusted for baseline tumor burden (SPD) and cell-of-origin (COO) subtype (interaction p-value < 0.05). The columns present the interaction hazard ratio (interaction HR), 95% confidence intervals (lower CI, upper CI), unadjusted p-values (interaction p-value), and Benjamini-Hochberg FDR-adjusted p-values (interaction FDR).

Gene	Interaction HR	CI lower	CI upper	Interaction PValue	Interaction FDR
KLRD1	1.45	1.14	1.85	0.002	0.049
FCN1	1.44	1.14	1.81	0.002	0.049
DUSP5	1.39	1.12	1.73	0.003	0.049
ADGRE1	1.38	1.11	1.71	0.003	0.049
CD19	0.74	0.60	0.92	0.006	0.053
LILRB2	1.48	1.12	1.94	0.006	0.053
CTSW	1.43	1.10	1.86	0.008	0.055
CD8A	1.35	1.08	1.68	0.008	0.055
CD244	1.43	1.09	1.86	0.009	0.055
PRF1	1.34	1.07	1.68	0.012	0.061
MAGEA4	1.34	1.07	1.68	0.012	0.061
CCL4	1.41	1.07	1.86	0.015	0.064
NCAM1	1.29	1.05	1.59	0.015	0.064
CD247	1.35	1.06	1.72	0.016	0.064
CD45RA	0.84	0.73	0.97	0.020	0.073
FCGR1A	1.27	1.03	1.56	0.023	0.079
C7	1.18	1.02	1.37	0.025	0.083
TNFRSF25	1.36	1.03	1.79	0.028	0.087
SHC2	1.35	1.03	1.78	0.031	0.093
XCL1/2	1.35	1.02	1.79	0.034	0.096
HSD11B1	1.25	1.01	1.54	0.038	0.100
NGFR	1.28	1.01	1.64	0.045	0.115
