# Supplementary material for: Baseline Tumor Transcriptional Features and Event-Free Survival After Anti-CD19 CAR T-Cell Therapy with Axicabtagene Ciloleucel in Large B-Cell Lymphoma: An Exploratory Reanalysis of the Randomized ZUMA-7 Trial
Source: Med Sci (Basel). 2026 Oct 13;14(6):671. doi: 10.3390/medsci14060671 (PMC13706688; doi:10.3390/medsci14060671)
Supplement: Supplementary file 1 [file medsci-14-00671-s001.zip › medsci-4564567-Tables.zip!/Supplementary_Table_S12_round2.pdf]

Supplementary Table S12. Survival analyses (Kaplan-Meier with log-rank test) and Cox proportional hazards analyses of the candidate genes presented in Figure 4A (derived from the multivariate model for axi-cel), evaluated in the axi-cel experimental arm, the standard-of-care (SOC) control arm of the ZUMA-7 study, and the TCGA-DLBC exploratory cohort.

Ensembl	Symbol	axi-cel P-Value	SOC P-Value	TCGA-DLBC P-Value	axi-cel Events	SOC Events	TCGA-DLBC Events	axi-cel FDR	SOC FDR	TCGA-DLBC FDR
ENSG00000138166	*DUSP5*	0.001	0.506	0.634	78	101	9	0.004	0.730	0.845
ENSG00000241106	*HLA-DOB*	0.009	0.310	0.915	78	101	9	0.017	0.730	0.918
ENSG00000156738	*MS4A1*	3.75E-05	0.786	0.472	78	101	9	4.50E-04	0.857	0.845
ENSG00000134539	*KLRD1*	0.006	0.337	0.266	78	101	9	0.014	0.730	0.646
ENSG00000170558	*CDH2*	0.043	0.133	0.269	78	101	9	0.043	0.730	0.646
ENSG00000140968	*IRF8*	0.035	0.574	0.597	78	101	9	0.041	0.730	0.845
ENSG00000085741	*WNT11*	0.005	0.474	0.036	78	101	9	0.014	0.730	0.431
ENSG00000177455	*CD19*	0.005	0.608	0.918	78	101	9	0.014	0.730	0.918
ENSG00000048462	*TNFRSF17*	0.010	0.170	0.759	78	101	9	0.017	0.730	0.911
ENSG00000149294	*NCAM1*	0.037	0.243	0.122	78	101	9	0.041	0.730	0.598
ENSG00000186265	*BTLA*	0.034	0.415	0.150	78	101	9	0.041	0.730	0.598
ENSG00000137507	*LRRC32*	0.019	0.975	0.616	78	101	9	0.029	0.975	0.845

Note: The CD45RA (*PTPRC*) isoform was excluded from the TCGA-DLBC analysis because precise isoform-level quantification is not available in the standard gene-level matrices of this exploratory dataset.
