# Supplementary material for: Baseline Tumor Transcriptional Features and Event-Free Survival After Anti-CD19 CAR T-Cell Therapy with Axicabtagene Ciloleucel in Large B-Cell Lymphoma: An Exploratory Reanalysis of the Randomized ZUMA-7 Trial
Source: Med Sci (Basel). 2026 Oct 13;14(6):671. doi: 10.3390/medsci14060671 (PMC13706688; doi:10.3390/medsci14060671)
Supplement: Supplementary file 1 [file medsci-14-00671-s001.zip › medsci-4564567-Tables.zip!/Supplementary_Table_S13_round2.pdf]

Supplementary Table S13. Survival (Kaplan-Meier and log-rank) and Cox proportional hazards analyses of candidate genes identified from significant treatment-by-gene interactions between axi-cel and SOC (Figure 4B), evaluated in the axi-cel and SOC arms and the TCGA-DLBC exploratory cohort.

Ensembl	Symbol	Axi-cel PValue	SOC PValue	TCGA-DLBC PValue	Axi-cel Events	SOC Events	TCGA-DLBC Events	Axi-cel FDR	SOC FDR	TCGA-DLBC FDR
ENSG00000134539	*KLRD1*	0.006	0.337	0.266	78	101	9	0.026	0.506	0.806
ENSG00000085265	*FCN1*	0.074	0.054	0.074	78	101	9	0.221	0.275	0.806
ENSG00000138166	*DUSP5*	0.001	0.506	0.634	78	101	9	0.012	0.651	0.806
ENSG00000174837	*ADGRE1*	0.002	0.011	0.612	78	101	9	0.016	0.095	0.806
ENSG00000177455	*CD19*	0.005	0.608	0.918	78	101	9	0.026	0.729	0.975
ENSG00000131042	*LILRB2*	0.692	0.098	0.993	78	101	9	0.733	0.278	0.993
ENSG00000172543	*CTSW*	0.278	0.186	0.252	78	101	9	0.455	0.371	0.806
ENSG00000153563	*CD8A*	0.588	0.076	0.444	78	101	9	0.661	0.275	0.806
ENSG00000122223	*CD244*	0.741	0.648	0.394	78	101	9	0.741	0.729	0.806
ENSG00000180644	*PRF1*	0.416	0.124	0.725	78	101	9	0.505	0.279	0.821
ENSG00000147381	*MAGEA4*	0.360	0.407	NA	78	101	0	0.505	0.563	NA
ENSG00000275302	*CCL4*	0.202	0.108	0.664	78	101	9	0.363	0.278	0.806
ENSG00000149294	*NCAM1*	0.037	0.243	0.122	78	101	9	0.134	0.397	0.806
ENSG00000198821	*CD247*	0.404	0.221	0.479	78	101	9	0.505	0.397	0.806
ENSG00000150337	*FCGR1A*	0.158	0.074	0.307	78	101	9	0.315	0.275	0.806
ENSG00000112936	*C7*	0.136	0.790	0.417	78	101	9	0.315	0.837	0.806
ENSG00000215788	*TNFRSF25*	0.151	0.871	0.596	78	101	9	0.315	0.871	0.806
ENSG00000129946	*SHC2*	0.421	0.002	0.442	78	101	9	0.505	0.039	0.806
