# Supplementary material for: Baseline Tumor Transcriptional Features and Event-Free Survival After Anti-CD19 CAR T-Cell Therapy with Axicabtagene Ciloleucel in Large B-Cell Lymphoma: An Exploratory Reanalysis of the Randomized ZUMA-7 Trial
Source: Med Sci (Basel). 2026 Oct 13;14(6):671. doi: 10.3390/medsci14060671 (PMC13706688; doi:10.3390/medsci14060671)
Supplement: Supplementary file 1 [file medsci-14-00671-s001.zip › medsci-4564567-Tables.zip!/Supplementary_Table_S14_round2.pdf]

Supplementary Table S14. MCP-counter marker gene availability and coverage mapping within the NanoString IO360 panel. Columns display the evaluated cell population, total marker genes defined by the original MCP-counter algorithm, number of markers successfully found in the NanoString IO360 dataset (Markers Found), percentage coverage (Percent Coverage), and the specific available marker genes detected.

**Population**	**Total Markers MCP**	**Markers Found**	**Percent Coverage (%)**	**Available Markers**
T cells	16	9	56.2	*CD28, CD3D, CD3G, CD5, CD6, CTLA4, ICOS, TNFRSF25, TRAT1*
CD8 T cells	1	1	100	*CD8B*
Cytotoxic lymphocytes	7	4	57.1	*CD8A, EOMES, GNLY, KLRD1*
B lineage	9	4	44.4	*CD19, CD79A, FCRL2, MS4A1*
NK cells	9	3	33.3	*KIR2DL3, KIR3DL1, NCR1*
Monocytic lineage	7	2	28.6	*CSF1R, FPR3*
Myeloid dendritic cells	6	0	0	
Neutrophils	15	3	20	*CEACAM3, CXCR2, TNFRSF10C*
Endothelial cells	33	11	33.3	*BCL6B, CDH5, CLEC14A, EDN1, FAM124B, KDR, MMRN2, MYCT1, PALMD, ROBO4, TIE1*
Fibroblasts	8	0	0	
