# Supplementary material for: Baseline Tumor Transcriptional Features and Event-Free Survival After Anti-CD19 CAR T-Cell Therapy with Axicabtagene Ciloleucel in Large B-Cell Lymphoma: An Exploratory Reanalysis of the Randomized ZUMA-7 Trial
Source: Med Sci (Basel). 2026 Oct 13;14(6):671. doi: 10.3390/medsci14060671 (PMC13706688; doi:10.3390/medsci14060671)
Supplement: Supplementary file 1 [file medsci-14-00671-s001.zip › medsci-4564567-Tables.zip!/Supplementary_Table_S15_round2.pdf]

Supplementary Table S15. Evaluation of estimated immune and stromal cell populations via MCP-counter deconvolution between sustained response and failure groups within the axi-cel-treated cohort of the ZUMA-7 study. Medians of estimated abundance scores, unadjusted p-values, and Benjamini-Hochberg False Discovery Rate (FDR) adjustments are displayed.

Cell type	Sustained Median	Failure Median	P-value	FDR
B lineage	11.37	10.95	0.004	0.029
Neutrophils	4.12	4.43	0.092	0.280
Endothelial cells	5.38	5.61	0.105	0.280
T cells	6.02	6.26	0.227	0.343
NK cells	4.06	4.14	0.242	0.343
CD8 T cells	6.45	6.52	0.291	0.343
Cytotoxic lymphocytes	6.73	6.73	0.300	0.343
Monocytic lineage	8.70	8.54	0.776	0.776
