# Supplementary material for: Baseline Tumor Transcriptional Features and Event-Free Survival After Anti-CD19 CAR T-Cell Therapy with Axicabtagene Ciloleucel in Large B-Cell Lymphoma: An Exploratory Reanalysis of the Randomized ZUMA-7 Trial
Source: Med Sci (Basel). 2026 Oct 13;14(6):671. doi: 10.3390/medsci14060671 (PMC13706688; doi:10.3390/medsci14060671)
Supplement: Supplementary file 1 [file medsci-14-00671-s001.zip › medsci-4564567-Tables.zip!/Supplementary_Table_S16_round2.pdf]

Supplementary Table S16. Evaluation of estimated immune and stromal cell populations via MCP-counter deconvolution between sustained response and failure groups within the standard-of-care (SOC) control arm of the ZUMA-7 study. Medians of estimated abundance scores, unadjusted p-values, and Benjamini-Hochberg False Discovery Rate (FDR) adjustments are displayed.

Cell type	Sustained Median	Failure Median	P-value	FDR
Monocytic lineage	9.36	8.87	0.023	0.115
B lineage	10.17	10.98	0.029	0.115
NK cells	4.85	4.33	0.067	0.152
Cytotoxic lymphocytes	7.37	6.85	0.076	0.152
Endothelial cells	5.95	5.57	0.210	0.337
CD8 T cells	7.03	6.39	0.273	0.364
T cells	6.48	6.52	0.477	0.545
Neutrophils	4.62	4.47	0.630	0.630
