# Supplementary material for: Baseline Tumor Transcriptional Features and Event-Free Survival After Anti-CD19 CAR T-Cell Therapy with Axicabtagene Ciloleucel in Large B-Cell Lymphoma: An Exploratory Reanalysis of the Randomized ZUMA-7 Trial
Source: Med Sci (Basel). 2026 Oct 13;14(6):671. doi: 10.3390/medsci14060671 (PMC13706688; doi:10.3390/medsci14060671)
Supplement: Supplementary file 1 [file medsci-14-00671-s001.zip › medsci-4564567-Tables.zip!/Supplementary_Table_S17_round2.pdf]

Supplementary Table S17. Proportional hazards diagnostics (scaled Schoenfeld residuals) for the multivariable Cox models of event-free survival in the axi-cel arm of ZUMA-7. Each model includes the candidate gene (continuous log_2 expression), baseline tumor burden (SPD, continuous) and cell of origin (COO), fitted on complete cases (n = 121 patients; 69 events). All 14 genes that met the screening rule (log-rank P < 0.05 and univariable Cox P < 0.05; Supplementary Table S8) are shown, including RORC, which did not reach P < 0.05 in the multivariable model and is therefore not part of the 13-gene panel (Supplementary Table S10). P-values derive from cox.zph (survival R package; Kaplan–Meier time transformation) for each term and for the global test; P < 0.05 indicates evidence against proportional hazards. In the pooled gene-by-treatment interaction models (Supplementary Table S11; 228 patients, 161 events), the treatment-arm and interaction terms showed evidence of non-proportional hazards in all 56 models (Schoenfeld P < 0.001), so interaction hazard ratios should be interpreted as averages over follow-up.

Gene	MultiCox HR (95% CI)	MultiCox P-value	Schoenfeld P (gene)	Schoenfeld P (SPD)	Schoenfeld P (COO)	Schoenfeld P (global)
DUSP5	1.324 (1.117–1.571)	0.001	0.744	0.376	0.067	0.156
HLA-DOB	0.779 (0.667–0.909)	0.002	0.486	0.363	0.133	0.264
MS4A1	0.835 (0.746–0.935)	0.002	0.611	0.418	0.156	0.270
KLRD1	1.322 (1.108–1.577)	0.002	0.206	0.361	0.075	0.102
CDH2	1.306 (1.103–1.548)	0.002	0.038	0.415	0.083	0.013
IRF8	0.775 (0.656–0.916)	0.003	0.488	0.399	0.116	0.179
WNT11	1.353 (1.082–1.691)	0.008	0.730	0.485	0.112	0.263
CD45RA	0.858 (0.765–0.962)	0.008	0.573	0.398	0.101	0.173
CD19	0.823 (0.710–0.954)	0.010	0.120	0.372	0.103	0.083
TNFRSF17	0.853 (0.757–0.963)	0.010	0.817	0.300	0.082	0.150
NCAM1	1.205 (1.038–1.398)	0.014	0.124	0.385	0.099	0.085
BTLA	0.837 (0.721–0.973)	0.020	0.588	0.341	0.130	0.213
LRRC32	1.194 (1.002–1.423)	0.048	0.689	0.396	0.118	0.235
RORC	1.181 (0.977–1.428)	0.086	0.014	0.393	0.100	0.020

Notes: HR, hazard ratio per log_2 unit; CI, confidence interval; SPD, sum of product diameters; COO, cell of origin. CDH2 showed evidence of non-proportionality for the gene term (P = 0.038) and in the global test (P = 0.013); its hazard ratio should be interpreted as an average effect over follow-up. Source: script_08_followup_schoenfeld.R.

Follow-up (reverse Kaplan–Meier, Followup_events.csv): axi-cel arm, 134 patients, 78 EFS events, median follow-up 23.7 months (IQR 19.5–24.5); SOC arm, 122 patients, 101 events, 21.6 months (IQR 18.3–26.8); complete cases for multivariable models: 121 (69 events) and 107 (92 events), respectively; TCGA-DLBC, 47 patients, 9 deaths, median follow-up 32.3 months.
