# Supplementary material for: Baseline Tumor Transcriptional Features and Event-Free Survival After Anti-CD19 CAR T-Cell Therapy with Axicabtagene Ciloleucel in Large B-Cell Lymphoma: An Exploratory Reanalysis of the Randomized ZUMA-7 Trial
Source: Med Sci (Basel). 2026 Oct 13;14(6):671. doi: 10.3390/medsci14060671 (PMC13706688; doi:10.3390/medsci14060671)
Supplement: Supplementary file 1 [file medsci-14-00671-s001.zip › medsci-4564567-Tables.zip!/Supplementary_Table_S18_round2.pdf]

Supplementary Table S18. Sensitivity analyses of the gene-by-treatment interaction for event-free survival in the pooled randomized ZUMA-7 population (56 candidate genes; 228 patients with complete covariates; 161 events). Primary model (as in Supplementary Table S11): Cox model with gene (continuous $\log_2$), treatment arm (SOC as reference), gene × arm interaction, SPD and COO. Arm-stratified model: gene, gene × arm, SPD and COO with strata(arm), which removes the proportional-hazards requirement for the treatment-arm main effect. Arm- and COO-stratified model: gene, gene × arm and SPD with strata(arm, COO). Interaction HRs are the ratio of the per-$\log_2$ HR in the axi-cel arm to that in the SOC arm. FDR values are Benjamini–Hochberg-adjusted across the 56 genes within each model. Genes are ordered by the arm-stratified P-value.

Gene	Primary: interaction HR (95% CI)	Primary P	Primary FDR	Arm-stratified: interaction HR (95% CI)	Arm-stratified P	Arm-stratified FDR	Schoenfeld P (gene × arm)	Arm + COO-stratified: interaction HR (95% CI)	Arm + COO-stratified FDR	Schoenfeld P (global)
DUSP5	1.39 (1.12–1.73)	0.003	0.049	1.43 (1.15–1.78)	0.001	0.047	0.733	1.50 (1.20–1.89)	0.028	0.978
KLRD1	1.45 (1.14–1.85)	0.002	0.049	1.47 (1.15–1.86)	0.002	0.047	0.215	1.47 (1.16–1.87)	0.035	0.462
FCN1	1.44 (1.14–1.81)	0.002	0.049	1.42 (1.13–1.79)	0.003	0.048	0.965	1.43 (1.12–1.82)	0.035	0.865
CD19	0.74 (0.60–0.92)	0.006	0.053	0.74 (0.60–0.91)	0.004	0.048	0.073	0.73 (0.59–0.90)	0.035	0.241
ADGRE1	1.38 (1.11–1.71)	0.003	0.049	1.36 (1.10–1.69)	0.004	0.048	0.980	1.38 (1.11–1.71)	0.035	0.568
LILRB2	1.48 (1.12–1.94)	0.006	0.053	1.46 (1.11–1.92)	0.006	0.058	0.983	1.56 (1.17–2.07)	0.035	0.969
CD244	1.43 (1.09–1.86)	0.009	0.055	1.42 (1.09–1.85)	0.009	0.058	0.069	1.42 (1.08–1.86)	0.043	0.366
CTSW	1.43 (1.10–1.86)	0.008	0.055	1.41 (1.09–1.84)	0.009	0.058	0.443	1.41 (1.09–1.82)	0.043	0.845
CD8A	1.35 (1.08–1.68)	0.008	0.055	1.34 (1.07–1.67)	0.009	0.058	0.145	1.33 (1.07–1.66)	0.043	0.421
CD45RA	0.84 (0.73–0.97)	0.020	0.073	0.83 (0.72–0.96)	0.010	0.059	0.633	0.80 (0.69–0.93)	0.035	0.550
CCL4	1.41 (1.07–1.86)	0.015	0.064	1.42 (1.08–1.87)	0.013	0.063	0.550	1.46 (1.10–1.93)	0.043	0.771
PRF1	1.34 (1.07–1.68)	0.012	0.061	1.33 (1.06–1.67)	0.014	0.063	0.350	1.38 (1.10–1.74)	0.039	0.644
CD247	1.35 (1.06–1.72)	0.016	0.064	1.35 (1.06–1.72)	0.015	0.063	0.802	1.35 (1.05–1.73)	0.049	0.860
NCAM1	1.29 (1.05–1.59)	0.015	0.064	1.29 (1.05–1.59)	0.017	0.069	0.175	1.29 (1.04–1.60)	0.049	0.530
C7	1.18 (1.02–1.37)	0.025	0.083	1.19 (1.03–1.38)	0.020	0.072	0.260	1.21 (1.05–1.41)	0.043	0.432
MAGEA4	1.34 (1.07–1.68)	0.012	0.061	1.31 (1.04–1.64)	0.021	0.072	0.421	1.34 (1.06–1.69)	0.049	0.337
FCGR1A	1.27 (1.03–1.56)	0.023	0.079	1.27 (1.03–1.56)	0.022	0.072	0.872	1.36 (1.09–1.68)	0.038	0.960
TNFRSF25	1.36 (1.03–1.79)	0.028	0.087	1.37 (1.04–1.81)	0.023	0.072	0.094	1.41 (1.07–1.86)	0.049	0.201
MS4A1	0.87 (0.75–1.01)	0.074	0.147	0.85 (0.73–0.99)	0.035	0.099	0.706	0.82 (0.70–0.97)	0.049	0.773
SHC2	1.35 (1.03–1.78)	0.031	0.093	1.35 (1.02–1.77)	0.035	0.099	0.046	1.40 (1.06–1.85)	0.049	0.106
HSD11B1	1.25 (1.01–1.54)	0.038	0.100	1.25 (1.01–1.54)	0.040	0.101	0.558	1.28 (1.03–1.58)	0.056	0.817
CDH2	1.23 (0.99–1.52)	0.065	0.136	1.25 (1.01–1.55)	0.043	0.101	0.152	1.36 (1.07–1.72)	0.043	0.020

IL32	1.27 (1.00–1.62)	0.052	0.122	1.28 (1.01–1.63)	0.044	0.101	0.878	1.33 (1.04–1.71)	0.051	0.798
NGFR	1.28 (1.01–1.64)	0.045	0.115	1.28 (1.01–1.64)	0.044	0.101	0.110	1.26 (0.98–1.60)	0.112	0.368
KLRB1	1.23 (1.00–1.52)	0.053	0.122	1.25 (1.00–1.54)	0.045	0.101	0.036	1.25 (1.00–1.55)	0.089	0.079
XCL1/2	1.35 (1.02–1.79)	0.034	0.096	1.32 (1.00–1.75)	0.049	0.105	0.200	1.30 (0.98–1.71)	0.112	0.105
C2	1.28 (1.00–1.63)	0.054	0.122	1.28 (1.00–1.63)	0.054	0.111	0.805	1.34 (1.04–1.73)	0.051	0.799
CCL21	1.11 (0.99–1.24)	0.078	0.151	1.11 (1.00–1.25)	0.060	0.120	0.528	1.15 (1.02–1.29)	0.049	0.370
MET	1.18 (0.97–1.43)	0.093	0.173	1.19 (0.98–1.44)	0.078	0.151	0.316	1.25 (1.02–1.52)	0.060	0.002
MAML3	1.25 (0.99–1.57)	0.061	0.131	1.22 (0.97–1.54)	0.083	0.151	0.019	1.22 (0.96–1.56)	0.156	0.315
CPA3	1.18 (0.96–1.46)	0.123	0.186	1.21 (0.98–1.49)	0.084	0.151	0.638	1.24 (1.00–1.54)	0.089	0.682
BTLA	0.85 (0.69–1.04)	0.118	0.183	0.84 (0.68–1.03)	0.096	0.163	0.622	0.85 (0.69–1.05)	0.188	0.664
NKG7	1.17 (0.97–1.40)	0.097	0.175	1.17 (0.97–1.40)	0.096	0.163	0.455	1.16 (0.97–1.39)	0.156	0.836
PDGFA	1.27 (0.94–1.71)	0.115	0.183	1.27 (0.94–1.71)	0.112	0.181	0.585	1.39 (1.01–1.91)	0.083	0.413
WNT11	1.18 (0.89–1.57)	0.238	0.334	1.26 (0.95–1.67)	0.113	0.181	0.619	1.34 (1.00–1.80)	0.094	0.788
NFAM1	1.25 (0.95–1.63)	0.106	0.183	1.24 (0.95–1.61)	0.117	0.182	0.753	1.24 (0.95–1.63)	0.156	0.847
HLA-DOB	0.90 (0.75–1.08)	0.264	0.344	0.86 (0.72–1.04)	0.125	0.183	0.391	0.83 (0.68–1.02)	0.116	0.372
CD14	1.23 (0.95–1.59)	0.112	0.183	1.22 (0.95–1.57)	0.127	0.183	0.543	1.35 (1.02–1.77)	0.068	0.919
EPCAM	1.16 (0.96–1.41)	0.130	0.187	1.16 (0.96–1.41)	0.128	0.183	0.019	1.15 (0.95–1.41)	0.215	0.028
CMKLR1	1.28 (0.93–1.77)	0.130	0.187	1.28 (0.93–1.76)	0.136	0.190	0.196	1.38 (0.99–1.92)	0.094	0.837
MAGEA1	1.21 (0.96–1.52)	0.108	0.183	1.19 (0.94–1.51)	0.139	0.190	0.836	1.22 (0.96–1.55)	0.156	0.025
CTAG1B	1.19 (0.88–1.59)	0.259	0.344	1.18 (0.88–1.59)	0.273	0.364	0.524	1.18 (0.87–1.61)	0.318	0.614
CXCL16	1.15 (0.86–1.55)	0.339	0.421	1.17 (0.88–1.58)	0.282	0.368	0.952	1.24 (0.92–1.68)	0.221	0.903
ITGB2	1.18 (0.89–1.56)	0.250	0.341	1.16 (0.87–1.53)	0.307	0.391	0.558	1.17 (0.88–1.56)	0.318	0.743
TNFRSF17	0.94 (0.78–1.12)	0.484	0.532	0.91 (0.77–1.09)	0.324	0.398	0.706	0.90 (0.75–1.07)	0.292	0.769
KIR3DL2	1.16 (0.89–1.50)	0.272	0.347	1.14 (0.88–1.48)	0.327	0.398	0.244	1.14 (0.88–1.49)	0.342	0.190
CSF1	1.13 (0.86–1.50)	0.381	0.463	1.14 (0.86–1.50)	0.364	0.426	0.058	1.17 (0.89–1.56)	0.318	0.139
LRRC32	1.09 (0.86–1.39)	0.485	0.532	1.12 (0.88–1.42)	0.365	0.426	0.627	1.15 (0.90–1.49)	0.318	0.811
CEBPB	1.12 (0.82–1.54)	0.465	0.532	1.15 (0.84–1.57)	0.382	0.437	0.924	1.24 (0.90–1.71)	0.243	0.840
CD68	1.14 (0.81–1.60)	0.460	0.532	1.14 (0.81–1.60)	0.465	0.521	0.353	1.21 (0.85–1.71)	0.318	0.813
SLC7A5	1.15 (0.82–1.61)	0.405	0.483	1.13 (0.81–1.58)	0.482	0.524	0.762	1.06 (0.76–1.50)	0.749	0.483
IRF8	0.96 (0.74–1.26)	0.782	0.811	0.91 (0.70–1.18)	0.487	0.524	0.522	0.88 (0.67–1.15)	0.377	0.624
RORC	1.04 (0.82–1.33)	0.730	0.772	1.08 (0.85–1.37)	0.524	0.554	0.025	1.09 (0.85–1.38)	0.525	0.058
CCND1	1.08 (0.78–1.49)	0.651	0.701	1.10 (0.80–1.53)	0.547	0.568	0.723	1.23 (0.88–1.71)	0.288	0.291

HLA-DRB5	0.99 (0.93–1.06)	0.831	0.846	0.99 (0.92–1.06)	0.748	0.762	0.463	0.99 (0.92–1.06)	0.756	0.802
SERPINA9	1.00 (0.91–1.11)	0.935	0.935	1.01 (0.91–1.12)	0.866	0.866	0.572	1.00 (0.90–1.12)	0.968	0.869

Notes: In the arm-stratified models, the gene × arm term showed evidence of non-proportional hazards in 5 of 56 models (MAML3, EPCAM, RORC, KLRB1 and SHC2), whereas the global test was significant in 55 of 56 models owing to the COO term ($P < 0.05$ in all 56); additional stratification by COO addressed this. Because candidates were prioritized on their association with outcome within one arm, all interaction estimates are subject to selection and are hypothesis-generating. HR, hazard ratio; CI, confidence interval; SPD, sum of product diameters; COO, cell of origin; SOC, standard of care; FDR, false discovery rate.
