# Supplementary material for: Baseline Tumor Transcriptional Features and Event-Free Survival After Anti-CD19 CAR T-Cell Therapy with Axicabtagene Ciloleucel in Large B-Cell Lymphoma: An Exploratory Reanalysis of the Randomized ZUMA-7 Trial
Source: Med Sci (Basel). 2026 Oct 13;14(6):671. doi: 10.3390/medsci14060671 (PMC13706688; doi:10.3390/medsci14060671)
Supplement: Supplementary file 1 [file medsci-14-00671-s001.zip › medsci-4564567-Tables.zip!/Supplementary_Table_S19_round2.pdf]

Supplementary Table S19. Time-split gene-by-treatment interaction for event-free survival in the pooled randomized ZUMA-7 population (228 patients). Follow-up was split at 6 months (128 events before and 33 events after 6 months), and period-specific gene and gene × arm terms were estimated in a single Cox model stratified by treatment arm and adjusted for SPD and COO, with robust (patient-clustered) standard errors. Shown are the four genes with interaction FDR < 0.05 in the primary model and CD19, NCAM1 and CD45RA (nominal or borderline interactions).

Gene	Interaction HR < 6 months (95% CI)	P (< 6 months)	Interaction HR ≥ 6 months (95% CI)	P (≥ 6 months)
KLRD1	1.33 (1.04–1.71)	0.024	2.18 (1.31–3.62)	0.003
DUSP5	1.36 (1.07–1.74)	0.013	1.82 (1.02–3.23)	0.041
FCN1	1.33 (1.04–1.71)	0.025	1.93 (1.21–3.07)	0.005
ADGRE1	1.26 (1.00–1.59)	0.051	2.47 (1.17–5.22)	0.018
CD19	0.71 (0.56–0.90)	0.004	0.89 (0.45–1.77)	0.738
NCAM1	1.24 (1.01–1.52)	0.041	1.56 (0.89–2.74)	0.122
CD45RA	0.84 (0.72–0.99)	0.034	0.77 (0.59–1.02)	0.071

Notes: Interaction HR, ratio of the per-$\log_2$ HR in the axi-cel arm to that in the SOC arm within each period. Estimates after 6 months are based on few events and are imprecise.
