# Supplementary material for: Baseline Tumor Transcriptional Features and Event-Free Survival After Anti-CD19 CAR T-Cell Therapy with Axicabtagene Ciloleucel in Large B-Cell Lymphoma: An Exploratory Reanalysis of the Randomized ZUMA-7 Trial
Source: Med Sci (Basel). 2026 Oct 13;14(6):671. doi: 10.3390/medsci14060671 (PMC13706688; doi:10.3390/medsci14060671)
Supplement: Supplementary file 1 [file medsci-14-00671-s001.zip › medsci-4564567-Tables.zip!/Supplementary_Table_S20_round2.pdf]

Supplementary Table S20. Panel-wide event-free survival screen in the axi-cel arm of ZUMA-7 (134 patients; 78 events). The log-rank test (median split) and univariable Cox regression (continuous log_2 expression) were applied to all 769 mRNA features of the NanoString IO 360 panel, with Benjamini–Hochberg correction across the whole panel, and compared with the FDR values computed within the 25 prioritized candidates (Supplementary Table S8). Shown are the 14 genes that entered the multivariable models; the eight core genes are listed first.

Gene	Log-rank P	Log-rank FDR (25 candidates)	Log-rank FDR (panel, 769)	Univariable HR	Univariable P	Univariable FDR (25 candidates)	Univariable FDR (panel, 769)
MS4A1	<0.001	0.001	0.029	0.847	0.002	0.014	0.295
CD19	0.005	0.024	0.399	0.813	0.004	0.014	0.295
TNFRSF17	0.010	0.031	0.479	0.865	0.012	0.023	0.391
HLA-DOB	0.009	0.031	0.479	0.796	<0.001	0.011	0.295
CD45RA	<0.001	0.006	0.175	0.868	0.006	0.015	0.295
DUSP5	<0.001	0.006	0.175	1.340	<0.001	0.006	0.174
KLRD1	0.006	0.024	0.399	1.289	0.002	0.014	0.295
WNT11	0.005	0.024	0.399	1.349	0.003	0.014	0.295
IRF8	0.035	0.062	0.555	0.804	0.005	0.015	0.295
BTLA	0.034	0.062	0.555	0.823	0.007	0.016	0.319
CDH2	0.043	0.068	0.555	1.178	0.035	0.054	0.620
NCAM1	0.037	0.062	0.555	1.158	0.045	0.059	0.622
LRRC32	0.019	0.045	0.555	1.181	0.038	0.055	0.622
RORC	0.020	0.045	0.555	1.226	0.024	0.040	0.548

Notes: Across the whole panel, one feature (MS4A1) reached log-rank FDR < 0.05 and none reached univariable Cox FDR < 0.05; no feature was FDR-supported by both tests. The lowest panel-wide univariable FDR was 0.174 (DUSP5). Because the 25 candidates were prefiltered for nominal differential expression between response groups, an outcome correlated with EFS, FDR values computed within that family are anti-conservative relative to the panel. The complete panel-wide results are provided in N2_panelwide_EFS_screen_axicel.csv. Source: script_09_round2_sensitivity.R.
